# Impact of mechanical ventilation on severe acute kidney injury in critically ill patients with and without COVID-19 – a multicentre propensity matched analysis

**DOI:** 10.1186/s13613-025-01424-4

**Published:** 2025-01-25

**Authors:** Fabian Perschinka, Timo Mayerhöfer, Teresa Engelbrecht, Alexandra Graf, Paul Zajic, Philipp Metnitz, Michael Joannidis

**Affiliations:** 1 Division of Intensive Care and Emergency Medicine, Department of Internal Medicine, Medical University Innsbruck, Anichstrasse 35, Innsbruck, 6020 Austria; 2 Institute of Medical Statistics, Center for Medical Data Science, Medical University of Vienna, Vienna, Austria; 3 Division of Anaesthesiolgy and Intensive Care 1, Department of Anaesthesiology and Intensive Care, Medical University of Graz, Graz, Austria

**Keywords:** Renal replacement therapy, Invasive mechanical ventilation, CARDS, Propensity score matched

## Abstract

**Background:**

Acute kidney injury (AKI) is common in critically ill patients and is associated with increased morbidity and mortality. Its complications often require renal replacement therapy (RRT). Invasive mechanical ventilation (IMV) and infections are considered risk factors for the occurrence of AKI. The use of IMV and non-invasive ventilation (NIV) has changed over the course of the pandemic. Concomitant with this change in treatment a reduction in the incidences of AKI and RRT was observed. We aimed to investigate the impact of IMV on RRT initiation by comparing critically ill patients with and without COVID-19. Furthermore, we wanted to investigate the rates and timing of RRT as well as the outcome of patients, who were treated with RRT.

**Results:**

A total of 8,678 patients were included, of which 555 (12.8%) in the COVID-19 and 554 (12.8%) in the control group were treated with RRT. In the first week of ICU stay the COVID-19 patients showed a significantly lower probability for RRT initiation (day 1: *p* < 0.0001, day 2: *p* = 0.021). However, after day 7 a reversed HR was found. In mechanically ventilated patients the risk was significantly higher for the initiation of RRT over the entire stay. While in non-COVID-19 patients this was a non-significant trend, in COVID-19 patients the risk for RRT was significantly increased. The median delay between initiation of IMV and requirement of RRT was observed to be longer in COVID-19 patients (5 days [IQR: 2–11] vs. 2 days [IQR: 1–5]). The analysis restricted to patients with RRT showed a significantly higher risk for ICU death in patients requiring IMV compared to patients without IMV.

**Conclusion:**

The analysis demonstrated that IMV as well as COVID-19 are associated with an increased risk for initiation of RRT. The association between IMV and risk of RRT initiation was given for all investigated time intervals. Additionally, COVID-19 patients showed an increased risk for RRT initiation during the entire ICU stay within patients admitted to an ICU due to respiratory disease. In COVID-19 patients treated with RRT, the risk of death was significantly higher compared to non-COVID-19 patients.

**Supplementary Information:**

The online version contains supplementary material available at 10.1186/s13613-025-01424-4.

## Introduction

Acute kidney injury (AKI) is a common complication in critically ill patients and is associated with increased morbidity and mortality. In the AKI-EPI study the requirement of renal replacement therapy (RRT) was as high as 13.5% in critically ill patients [1], while reported rates in patients admitted because of sepsis were even higher [2]. Mortality rates in patients treated with RRT are up to 44% [3].

Sepsis and septic shock are among the most common causes for AKI [4], but the impact of severe viral infections is still unclear. During the COVID-19 pandemic relatively low rates of AKI were reported initially [5]. However, later cohort studies in critically ill patients and meta-analysis showed normal AKI incidences for critically ill patients with relatively high rates of RRT [6, 7].

Since angiotensin converting enzyme 2 (ACE2) receptors are present in the proximal tubular cells, a direct impact of SARS-CoV-2 on the kidneys seemed plausible [8–11]. However autopsy and biopsy studies indicated a rather multifactorial aetiology of AKI [12–15]. Since in respiratory failure COVID-19 primarily affects the lungs, especially in critically ill, lung-kidney interactions as a trigger of AKI may be of particular interest. This is especially true for severe respiratory failure since hypoxemia itself may interfere with renal blood flow [16].

A possible modifiable risk factor for AKI in critically ill patients may be invasive mechanical ventilation (IMV), which was shown in a meta-analysis [17]. At the beginning of the pandemic IMV rates were relatively high because of uncertainty and reports about rapid respiratory deterioration [18, 19]. A high post end-expiratory pressure (PEEP) leads to an increased intrathoracic and intraabdominal pressure and a consequently impaired renal perfusion [20]. As knowledge grew [21], non-invasive ventilation (NIV) strategies became more prominent. Concomitant with this change in treatment a reduction in the incidence of AKI and RRT was observed in cohort studies [22, 23].

The aim of this study was to investigate the association of IMV on RRT by comparing critically ill patients with and without COVID-19 over the first two years of pandemic. Furthermore, we wanted to investigate the rates and timing of RRT as well as the outcome of patients treated with RRT.

## Methods

### Study design and setting

This retrospective, multicentre registry study was based on prospectively gathered data collected by the Austrian Centre for Documentation and Quality Assurance in Intensive Care (ASDI). This database involves 154 intensive care units (ICUs) in Austria and comprised anonymised data from 114,634 patients in the period from January 1, 2020 to December 31, 2021. The registry contains data about demographics, hospital stay, reason for ICU admission (a detailed list of the categories is presented in the Electronic Supplemental Material, ESM Table 1), scores (Simplified Acute Physiology Score [SAPS 3] [24] and Simplified Therapeutic Intervention Scoring System 28 [TISS28] [25]) as well as patients outcome. Data about ICU therapy provided by ASDI contains the following variables: COVID-19 (binary: Yes/No), time to ICU-mortality as well as time to airway, ventilation mode and renal support for each day on the ICU per patient. Patients were defined to have IMV if they had an endotracheal tube or tracheal cannula at the same time as assisted breathing, Biphasic Positive Airway Pressure (BIPAP), controlled ventilation or high-frequency (HF)-Ventilation in the daily documentation. Hemodialysis (acute), Hemofiltration (intermittent/continuous) and Hemodiafiltration were defined as RRT. A detailed description of the ASDI database was published previously [26]. All analyses for this study were based on datasets derived from this database.

### Participants

All non-surgical, adult patients who had been discharged in the years 2020 or 2021 after an ICU stay of more than 48 h were included in this analysis. Patients admitted to an intermediate care unit (IMCU) were excluded. Additionally, patients were not considered for inclusion if daily documented variables of the ICU stay, like airway, ventilation mode or renal support, were not available during the entire ICU stay, although the missing values of single days did not lead to exclusion.

Patients were classified as COVID-19 patients, if a patient was admitted to ICU with COVID-19 or were diagnosed with COVID-19 during the ICU stay.

To aim for covariate balance a 1:1 nearest neighbour propensity score matching without replacement was performed by using the R-package Matchlt [27]. The propensity score was estimated using logistic regression of COVID-19 on the covariates IMV (ever prior to RRT), SAPS 3, age and sex. Patients whose propensity score difference was larger than 0.001 (caliper width of 0.001) were not paired. Only patients with a match were included in the analyses.

### Ethic declaration

The study (‘Auswirkung Invasiver Beatmung auf Akutes Nierenversagen bei Kritisch Kranken mit und ohne COVID-19’) was approved by the ethics committee of the Medical University of Graz (36–275 ex 23/24) on 4th April 2024. The Institutional review board refrain the need for informed consent since analyzed data were anonymized and no additional interventions were performed.

### Statistical analysis

Descriptive statistics were calculated for continuous variables (mean, standard deviation, first quartile, median, third quartile) and categorical variables (numbers, percent) for all patients as well as separately for the compared groups.

Main analysis: To investigate the effect of COVID-19 additionally to IMV on the need of RRT, a Cox proportional hazards model for time to RRT was performed. Due to the non-proportional hazard behaviour, COVID-19 was split into more categories: on the first day, the second day, the rest of the first week (day 3 to 7), the second week, the third week and the rest of the stay. Mechanical ventilation was split into two groups: IMV for a maximum of 7 days during the ICU stay and IMV for more than seven days. Both were used as time-dependent covariates. As further covariables age, sex and SAPS 3 were included in the model with ICU-ID as clustering variable. Age was categorized into four specified groups: 18–59, 60–69, 70–79 and 80 + years. SAPS 3 Score was categorized into three groups, based on tertiles. Patients dying on an ICU before the start of RRT were censored at the day of death. The model assumed that possible influences of IMV on RRT are time-delayed by at least one day, i.e. patients that had IMV and RRT on the first ICU day were allocated to the “No IMV”-group.

As sensitivity analysis, the main model was additionally evaluated separately for the two groups (COVID-19 and non-COVID-19) excluding the group factor from the model.

A further sensitivity analysis was carried out due to the different proportion of patients admitted to an ICU because of the main diagnosis of “respiratory disease” in the overall (matched) cohort. In this subgroup analysis only patients with the main diagnosis “respiratory disease” were considered in both groups in order to minimise the effects of possible differences in the treatments. Furthermore, sensitivity analyses were also performed with BMI as an additional matching covariate and with each comorbidity included in the SAPS 3 instead of SAPS 3 score as a matching covariate.

Another Cox proportional hazards model was performed to investigate the effect of IMV on the ICU survival. In this model, the same covariates were used as in the main model with the exception of IMV: if IMV and RRT were documented concurrently on at least one day during their stay, patients were considered invasively mechanically ventilated (binary 0/1). Time in the ICU until the start of RRT was used as additional covariate. For sensitivity analysis a similar model was performed for the combined outcome RRT or ICU-death. The above described variables included in the analysis remained the same.

P-values smaller than 0.05 were considered as statistically significant. All analyses were performed using R version 4.3.1. The used R packages are listed in the ESM Table 2.

## Results

### Patient characteristics

A total of 28,572 patients were included, of whom 4581 patients had COVID-19. The baseline characteristics of all (unmatched) patients are presented in ESM Table 3. After propensity score matching 4339 patients were assigned to each group (a flowchart can be found in the ESM Fig. 1). The overall cohort (after matching) had a median age of 66 years and was predominantly male (68.1%). Baseline characteristics can be found in Table 1.

**Table 1:** Baseline characteristics

	Overall (*n* = 8678)	Non-COVID-19 (*n* = 4339)	COVID-19 (*n* = 4339)
Age	66.0 (57.0–74.0)	67.0 (57.0–75.0)	66.0 (56.0–74.0)
Sex			
Female	2767 (31.9%)	1367 (31.5%)	1400 (32.3%)
Male	5911 (68.1%)	2972 (68.5%)	2939 (67.7%)
SAPS 3	55.0 (48.0–62.0)	55.0 (47.0–62.0)	55.0 (48.0–62.0)
**Main diagnosis**			
Metabolic disease	159 (1.8%)	137 (3.2%)	22 (0.5%)
Respiratory disease	4875 (56.2%)	1370 (31.6%)	3505 (80.8%)
Cardiovascular disease	671 (7.7%)	604 (13.9%)	67 (1.5%)
Shock	163 (1.9%)	144 (3.3%)	19 (0.4%)
Renal disease	251 (2.9%)	198 (4.6%)	53 (1.2%)
Neurologic disease	458 (5.3%)	402 (9.3%)	56 (1.3%)
Sepsis	160 (1.8%)	137 (3.2%)	23 (0.5%)
Trauma (not operated)	273 (3.1%)	255 (5.9%)	18 (0.4%)
Gastrointestinal disease	185 (2.1%)	174 (4.0%)	11 (0.3%)
Hematologic disease	19 (0.2%)	16 (0.4%)	3 (0.1%)
Medical	89 (1.0%)	50 (1.2%)	39 (0.9%)
Other	1375 (15.8%)	852 (19.6%)	523 (12.1%)
**Length of stay and mortality**			
ICU LOS	8 (5–16)	6 (4–13)	10 (6–18)
Hospital LOS	19 (11–31)	17 (10–31)	20 (13–32)
ICU mortality	1970 (22.7%)	692 (16.0%)	1278 (29.5%)
Hospital mortality	2578 (30.1%)	1078 (25.2%)	1500 (34.9%)
SAPS 3 observed/expected mortality ratio	1.04 (1.01–1.07)	0.87 (0.83–0.91)	1.20 (1.16–1.25)
**Most invasive airway (before RRT)**			
No support	117 (1.3%)	87 (2.0%)	30 (0.7%)
O2 (mask or nasal cannula)	1000 (11.5%)	803 (18.5%)	197 (4.5%)
NHFO	234 (2.7%)	57 (1.3%)	177 (4.1%)
NIV (mask or helmet)	2013 (23.2%)	690 (15.9%)	1323 (30.5%)
Endotracheal tube	4012 (46.2%)	2003 (46.2%)	2009 (46.3%)
Tracheal Cannula	1096 (12.6%)	553 (12.7%)	543 (12.5%)
Missing	7 (0.1%)	7 (0.2%)	0 (0.0%)
**Most invasive ventilation mode (before RRT)**			
Spontaneous breathing	1411 (16.3%)	988 (22.8%)	423 (9.7%)
CPAP	1243 (14.3%)	425 (9.8%)	818 (18.9%)
Assisted	1151 (13.3%)	521 (12.0%)	630 (14.5%)
BIPAP	1706 (19.7%)	907 (20.9%)	799 (18.4%)
HF-Ventilation	60 (0.7%)	15 (0.3%)	45 (1.0%)
Controlled	2879 (33.2%)	1325 (30.5%)	1554 (35.8%)
Controlled & HF-Ventilation	18 (0.2%)	11 (0.3%)	7 (0.2%)
Missing	11 (0.1%)	8 (0.2%)	3 (0.1%)
**Differences in initiation of RRT and IMV**			
RRT performed	1109 (12.8%)	554 (12.8%)	555 (12.8%)
RRT and IMV simultaneously	805 (9.3%)	319 (7.4%)	486 (11.2%)
RRT initiation after IMV	634 (7.3%)	249 (5.7%)	385 (8.9%)
Initiation of RRT and IMV on the same day	98 (1.1%)	44 (1.0%)	54 (1.2%)
RRT initiation before IMV	73 (0.8%)	26 (0.6%)	47 (1.1%)
RRT initiation after IMV (days)	3.0 (1.0–8.0)	2.0 (1.0–5.0)	5.0 (2.0–11.0)
RRT initiation before IMV (days)	2.0 (1.0–4.0)	3.0 (2.0–5.8)	2.0 (1.0–4.0)
Sum of the days of RRT	5.0 (3.0–11.0)	5.0 (3.0–8.0)	6.0 (3.0–12.0)
Sum of the days of IMV	2.0 (0.0–9.0)	2.0 (0.0–6.0)	4.0 (0.0–12.0)

Legend: SAPS 3 - Simplified acute physiology score 3; ICU LOS – Intensive care unit length of stay; Hospital LOS - Hospital length of stay; NIV – Non-invasive ventilation; CPAP – Continuous positive airway pressure; NHFO – Nasal high-flow oxygen; BIPAP - Biphasic positive airway pressure; HF-Ventilation – High-frequency ventilation; IMV – Invasive mechanical ventilation; RRT – Renal replacement therapy

### Respiratory support

Overall, most patients were admitted to the ICU due to respiratory disease (56.2%). Whereas low-flow oxygen therapy was applied more often in the non-COVID-19 group, continuous positive airway pressure (CPAP) applied by mask or helmet was used approximately twice as frequently in the COVID-19 group. IMV requiring endotracheal intubation was the most frequently used respiratory support modality before initiation of RRT both in the non-COVID-19 group (46.2%) and in the COVID-19 group (46.3%). The majority of invasively ventilated patients was treated with BIPAP (20.9% vs. 18.4%) or controlled ventilation (30.5% vs. 35.8%).

**Fig. 1:**
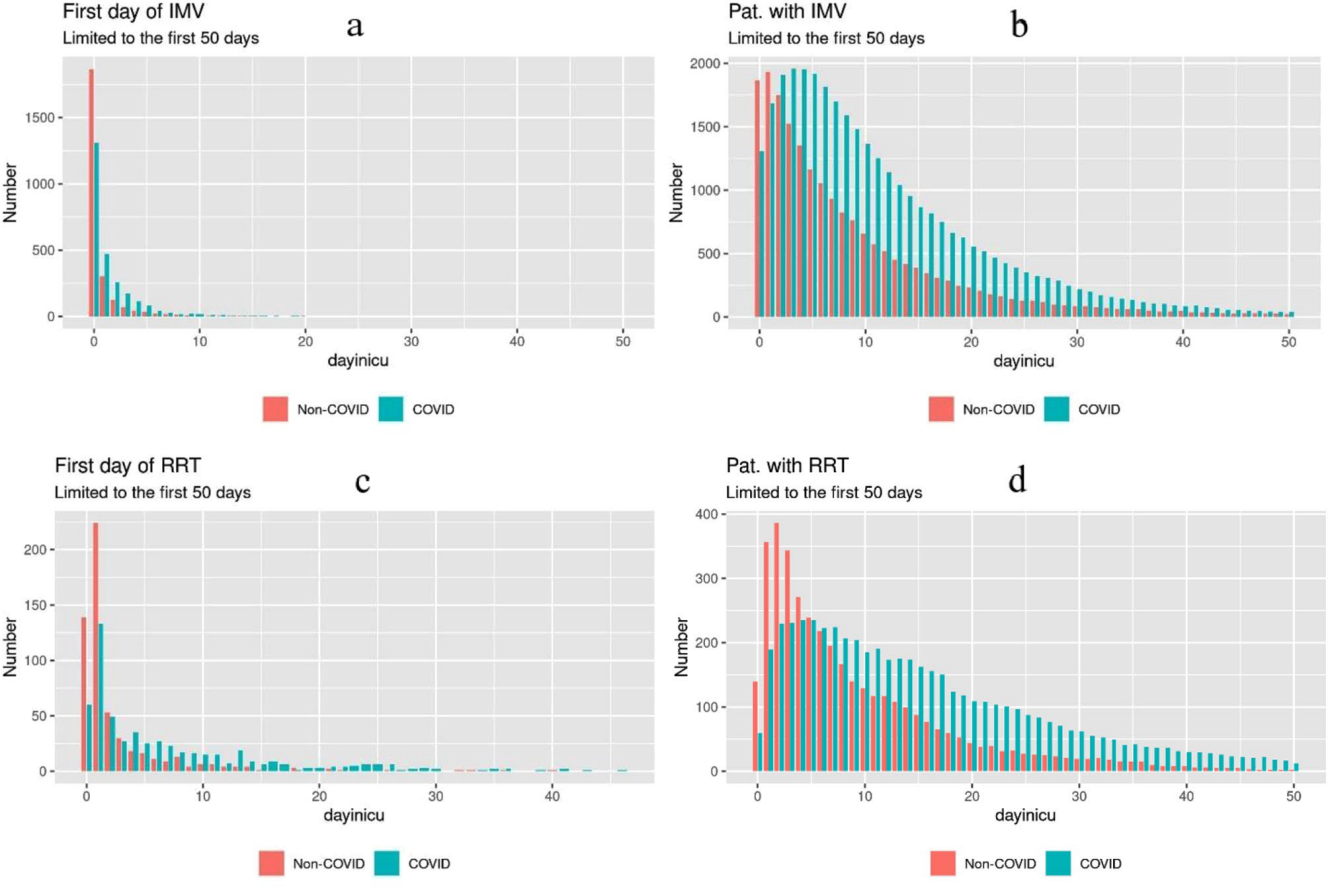
Course of initiation and total numbers of IMV and RRT

In most of the patients IMV was initiated at the beginning of ICU stay (day 0). Due to the higher number of patients starting IMV on the subsequent days (day 1–10) in COVID-19 patients, the peak of invasively mechanical ventilated patients occurred later in this group (Fig. 1A and B).

### Risk for RRT

**Fig. 2:**
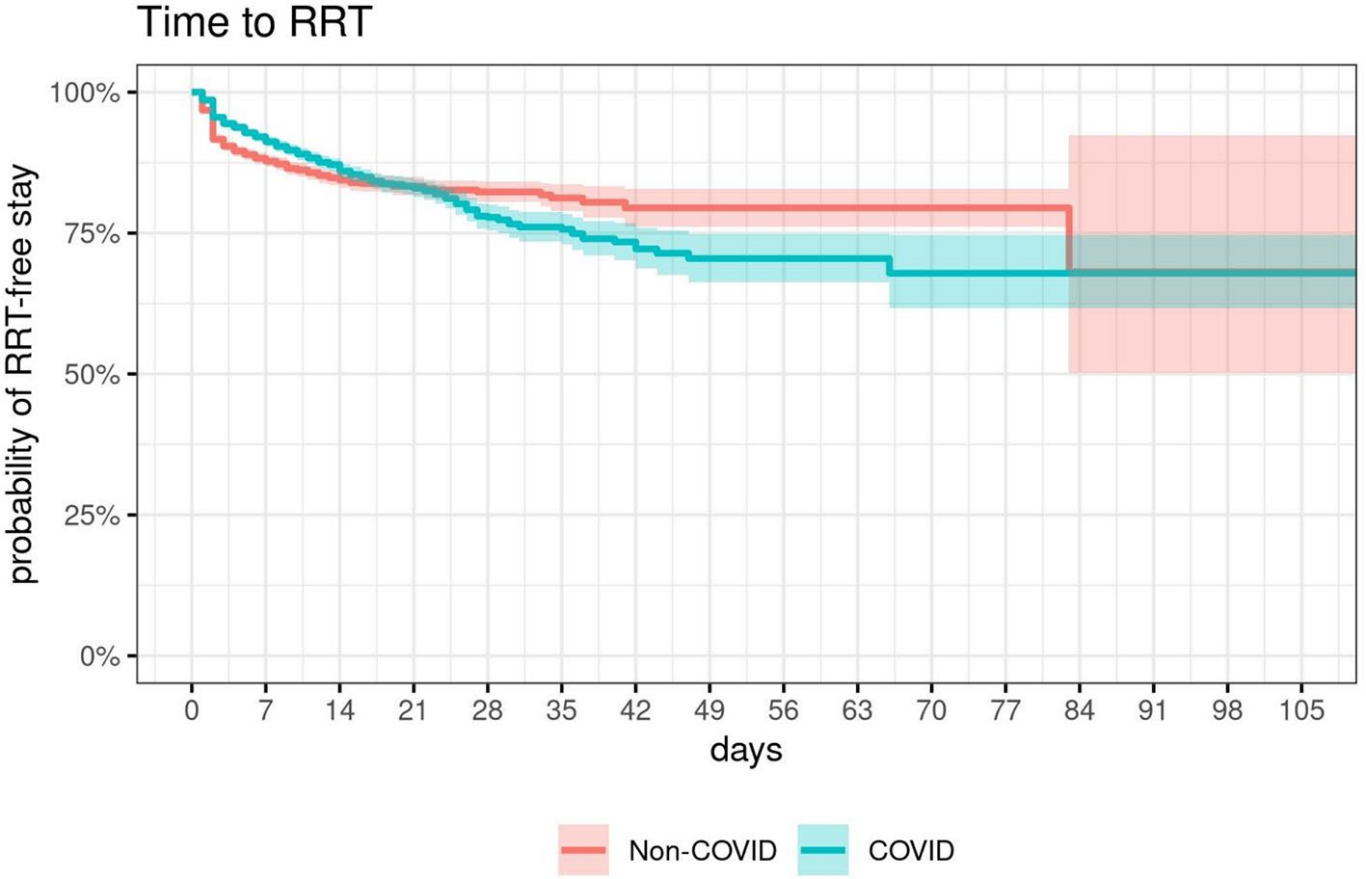
RRT-free days of COVID-19 patients and non-COVID-19 patients

**Fig. 3:**
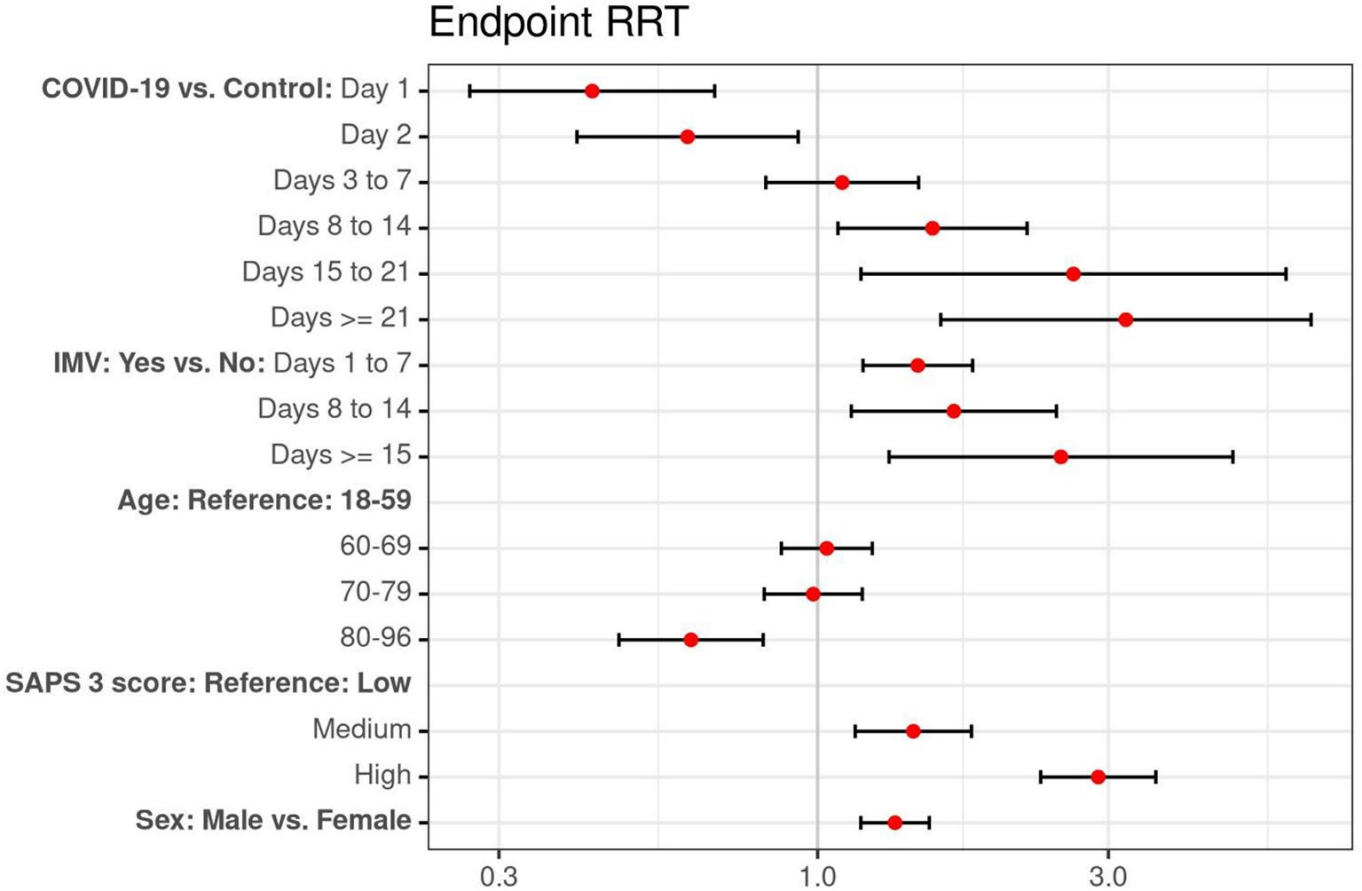
Hazard ratio of RRT initiation

In total 1109 (12.8%) patients were treated with RRT during their ICU stay, of whom 555 (12.8%) belonged to the COVID-19 group and 554 (12.8%) to the control group. The Kaplan-Meier plot (Fig. 2) shows the probability of RRT-free ICU stays for COVID-19 and non-COVID-19 patients. To correct for the non-proportional hazards (crossing curves), the COVID-19 group factor was split to evaluate the observed time trend. On day 1 and 2 of their ICU stay COVID-19 patients showed a significantly lower probability to start RRT as compared to non-COVID-19 patients (day 1: *p* < 0.0001, day 2: *p* = 0.021). However, after day 7 a reversed significant HR was found: the longer the patients stayed, the higher the risk for RRT requirement was in COVID-19 compared to non-COVID-19 patients. Patients infected with SARS-CoV-2 and an ICU stay longer than 20 days exhibited a 3.20-fold higher hazard for initiation of RRT (Fig. 3 and 95% CI: 1.59–6.45).

Rates of RRT remained constant and quite similar for both groups over calendar time, with the only exception of Q2 in 2020, where the proportion of patients treated with RRT increased transiently in COVID-19 patients (approximately 25%) (ESM Fig. 2).

Mechanically ventilated patients carried a significantly higher risk for RRT observed for all investigated periods (Day 1 to 7; Day 7 to 14; Day ≥ 15) (Fig. 3). The association between IMV and increased risk of RRT was observed in both COVID-19 and non-COVID-19 patients groups, if analyzed separately. This persisted across all intervals except from day 1 to day 7 in non-COVID-19 patients. While in non-COVID-19 patients this pattern was a non-significant trend, in COVID-19 patients a significantly higher risk for the requirement of RRT for invasively mechanical ventilated patients was observed for all investigated time intervals (ESM Table 4a and b).

In the COVID-19 group, 87.6% required IMV at the same time as RRT, compared with 57.6% in the non-COVID-19 group. In both groups, in about 1% of the patients, IMV and RRT were started on the same day. In approximately 80% of the patients, RRT was initiated at least one day after the start of IMV. The median delay between initiation of IMV and subsequent requirement of RRT was longer in COVID-19 patients (5 days; IQR: 2–11) compared to the non-COVID-19 group (2 days; IQR: 1–5). No clinically relevant difference was observed in the duration of RRT (COVID-19: 6 days [IQR: 3–12]; non-COVID-19: 5 days [IQR: 3–8]).

Adding body mass index (BMI) as additional covariate for propensity score matching showed an elevated risk for RRT initiation in the COVID-19 cohort from day 7 onwards as observed in the main analysis (ESM Table 5). Considering each comorbidity of the SAPS 3 as covariate instead of the SAPS 3 points also demonstrated a higher risk of RRT initiation in the COVID-19 cohort from day 8 onwards (ESM Table 6). In both sensitivity analyses the risk for RRT initiation in invasively mechanically ventilated patients was increased during the entire stay.

The main analyses were additionally performed with the combined endpoint RRT or death. The pattern obtained was similar to the main analysis, but the HRs increased with age group. The results are presented in the ESM Tables 7 and ESM Fig. 3 as well as 4a and 4b.

### Mortality

Both crude ICU and hospital mortality were higher in COVID-19 patients than in non-COVID-19 patients (29.5% vs. 16.0% and 34.9% vs. 25.2%, respectively). When comparing mortalities expected by the respective SAPS 3 scores, the COVID-19 cohort showed excess mortality. Standardised (observed-to-expected) mortality ratios were 1.20 (95% CI 1.16–1.25) in COVID-19 patients compared to 0.87 (95% CI 0.83–0.91) in the non-COVID-19 patients.

**Fig. 4:**
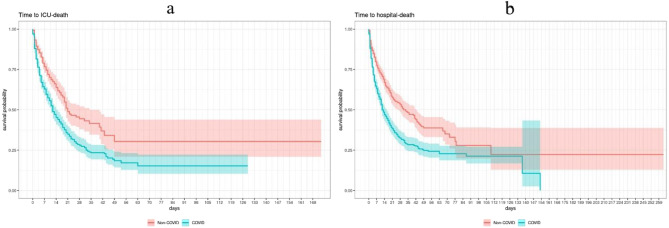
ICU mortality and hospital mortality of COVID-19 patients and non-COVID-19 patients treated with RRT

When RRT was initiated, COVID-19 patients had a significantly lower survival probability. The Kaplan-Meier curves illustrate the course of ICU and hospital mortality after starting RRT (first day of RRT is day 0) (Fig. 4a and b). This was also true in each different age group (ESM Fig. 5a and b).

The Cox proportional hazards model restricted to patients with RRT showed a significantly higher hazard for ICU-death in COVID-19 patients compared to the control group and a significantly higher hazard in patients requiring IMV compared to patients without IMV (Table 2).

**Table 2:** Cox proportional hazards for ICU-death restricted to RRT patients

		HR	Lower	Upper	*p*-value
COVID-19 vs. Control		1.53	1.21	1.94	**< 0.0001**
IMV concurrent with RRT: Yes vs. No		1.82	1.33	2.48	**< 0.0001**
Days in ICU before the start of RRT		1.00	0.99	1.01	0.643
Age: Reference: 18–59					
	60–69	1.57	1.14	2.18	**0.006**
	70–79	1.63	1.15	2.31	**0.006**
	80–96	2.10	1.32	3.33	**0.002**
SAPS 3 score: Reference: Low					
	Medium	0.98	0.75	1.29	0.896
	High	1.31	1.00	1.71	0.052
Sex: Male vs. Female		0.98	0.81	1.19	0.817

Legend: IMV – invasive mechanical ventilation; RRT – renal replacement therapy; ICU – Intensive Care Unit; SAPS 3 - Simplified Acute Physiology Score 3

### Sensitivity analysis restricted to patients with respiratory disease

In order to reduce bias due to varying reasons for ICU admission, a sensitivity analysis was performed restricted to patients with respiratory disease as main diagnosis in both the non-COVID-19 and in the COVID-19 group.

Baseline characteristics were similar to the overall cohort. Furthermore, the restriction to respiratory diseases balanced differences in the respiratory support modalities between the COVID-19 group and the non-COVID-19 group, especially the usage of CPAP therapy (ESM Table 8).

**Fig. 5:**
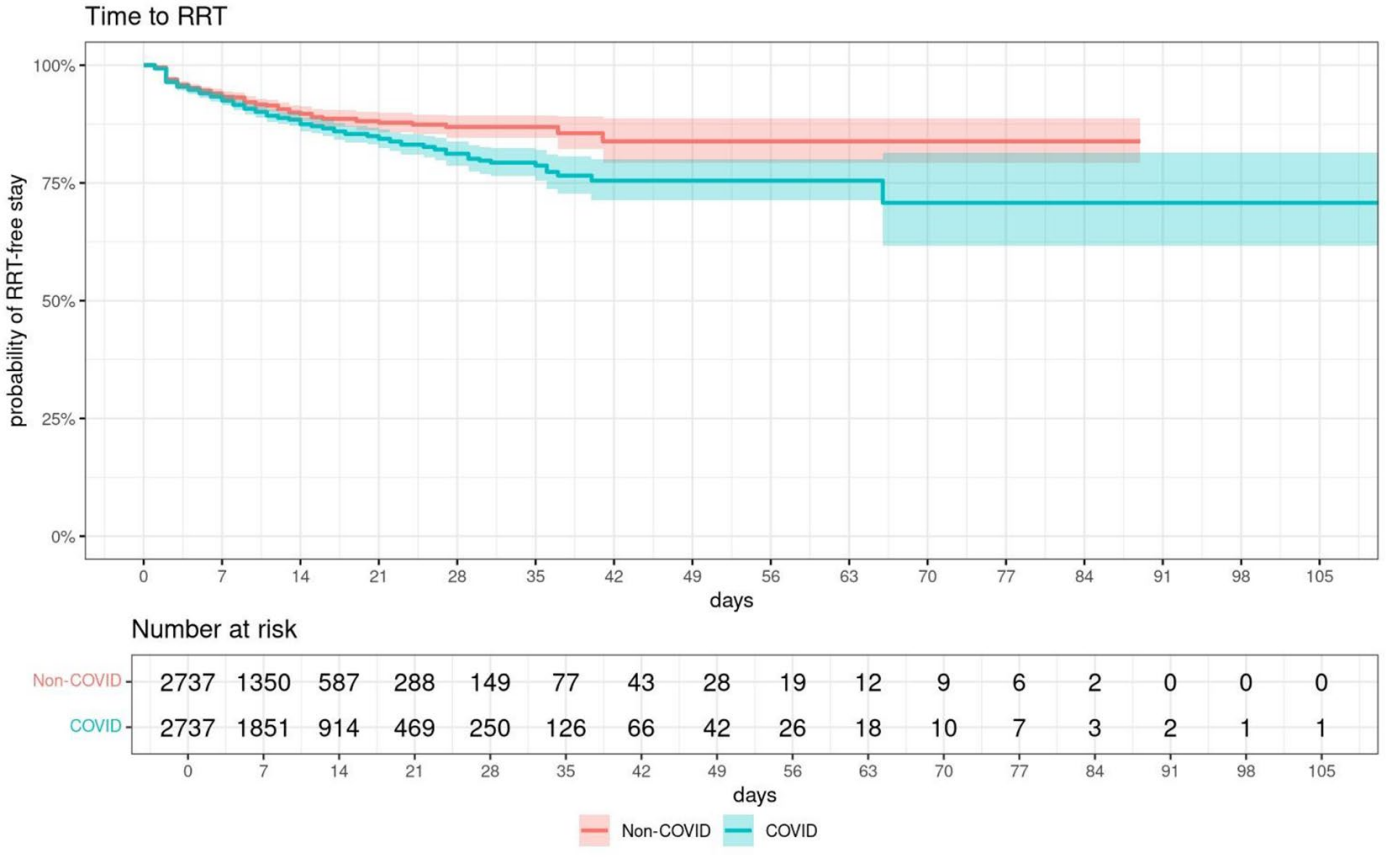
RRT-free stay of COVID-19 patients and non-COVID-19 patients admitted due to respiratory disease

Overall, in patients admitted to an ICU due to respiratory disease, 515 (9.4%) required RRT during their ICU stay. However, in contrast to the overall cohort, no crossing Kaplan-Meier curves were seen. COVID-19 patients showed a trend for a higher risk for RRT initiation during the entire ICU stay resulting in a more rapid decrease in the probability of RRT-free stay as compared to non-COVID-19 patients, which became statistically significant only after 21 days. Additionally, a significantly increased risk for RRT initiation (Days 1 to 7: 2.34 [CI: 1.79–3.06]; Days 8 to 14: 2.84 [CI: 1.78–4.54]; Days > = 15: 3.51 [CI: 1.33–9.22]) was seen in invasively mechanical ventilated patients (Fig. 5; Table 3).

**Table 3:** Cox proportional hazards for RRT initiation restricted to respiratory diseases

		HR	Lower	upper	*p*-value
COVID-19 vs. Control					
	Day 1	1.50	0.71	3.18	0.291
	Day 2	1.29	0.78	2.13	0.325
	Days 3 to 7	1.21	0.85	1.73	0.298
	Days 8 to 14	1.57	0.94	2.61	0.084
	Days 15 to 21	1.67	0.81	3.44	0.161
	Days > = 21	4.08	1.46	11.41	**0.007**
IMV: Yes vs. No					
	Days 1 to 7	2.34	1.79	3.06	**< 0.0001**
	Days 8 to 14	2.84	1.78	4.54	**< 0.0001**
	Days > = 15	3.51	1.33	9.22	**0.011**
Age: Reference: 18–59					
	60–69	0.93	0.71	1.23	0.628
	70–79	0.83	0.64	1.06	0.140
	80–95	0.44	0.30	0.65	**< 0.0001**
SAPS 3 score: Reference: Low					
	Medium	1.58	1.23	2.03	**< 0.0001**
	High	3.13	2.33	4.20	**< 0.0001**
Sex: Male vs. Female		1.56	1.29	1.88	**< 0.0001**

IMV – invasive mechanical ventilation; RRT – renal replacement therapy; SAPS 3 - Simplified Acute Physiology Score 3

 Whereas invasively mechanical ventilated COVID-19 patients exhibited a significantly increased risk for RRT initiation (HR days 1–7: 2.71 [CI: 1.93–3.81]; HR days 8–14: 3.05 [CI: 1.79–5.20]; HR days ≥ 15: 4.38 [1.74–11.0]) (ESM Table 9),  no difference in the probability of RRT free days between the COVID-19 group and the non-COVID-19 was observed in patients who did not receive invasive mechanical ventilation (ESM Fig. 6).

If RRT was initiated, the probability of ICU and hospital death was higher in the COVID-19 group in this sensitivity analysis (ESM Fig. 7). The pattern seen in this sensitivity analysis was similar to the main analysis. Adding BMI as an additional covariate for propensity score matching did not affect the association of IMV with RRT (ESM Table 10).

## Discussion

AKI requiring RRT is an important issue in both critically ill COVID-19 and non-COVID-19 patients. This multicenter registry study shows that IMV is significantly associated with the initiation of RRT, especially with prolonged use. In addition, significant differences in the risk of RRT requirement were observed between COVID-19 patients and non-COVID-19 patients. After initiating RRT a shorter time to ICU death and a higher rate of mortality was seen in the COVID-19 group.

### IMV as a risk factor for RRT in COVID-19 and non-COVID-19 patients

In our analysis, IMV was significantly associated with the initiation of RRT in COVID-19 patients, while in the non-COVID-19 group a trend towards higher hazard ratios was seen. IMV has already been demonstrated as a risk factor in several studies [7, 17]. Wealso observed a difference between COVID-19 and non-COVID-19 patients. The reasons for this difference may be multifactorial; on the one hand, the use of extraordinary high ventilation pressures which are sometimes necessary in COVID-19 acute respiratory distress syndrome (CARDS) [28] could have been responsible for this finding. Since high ventilation pressures lead to a decline in cardiac output and a reduction in renal blood flow as well as venous congestion [29], a negative impact on kidney perfusion seems a likely consequence. This assumption is strengthened by our observation that the majority of RRT treatments were initiated after the start of IMV, especially in the COVID-19 group. In a seminal study evaluating mechanical ventilation strategies, the kidney was shown to be the most affected organ by “conventional” ventilation compared to protective ventilation which turned out favourable [30]. In addition to hemodynamic effects of IMV, systemic release of inflammatory mediators released by the injured lung could have an impact on the kidneys [20]. Thus, duration of IMV may play a major role in the development of AKI and RRT requirement, as we could demonstrate for both non-COVID-19 and COVID-19 patients in the present study, with the mean duration of IMV observed to be longer in COVID-19 patients. It must, however, be conceived that the non-COVID-19 and the COVID-19 cohort compared in our main analysis though well matched with regards to IMV and severity of disease showed some differences in the main diagnosis at ICU admission, especially with the rates of respiratory disease. To correct for this potential bias we performed a sensitivity analysis including patients with respiratory diseases only. In this sensitivity analysis the HR for RRT associated with IMV was even higher and further increasing with duration of IMV. It may be assumed that this is caused by inflammation and associated damage of the kidneys through proposed lung-kidney interactions [20]. Previously published reports showed that the release of cytokines caused by the biotrauma of the lungs in ARDS is associated with the occurrence of AKI [31]. However, the impact of ventilation cannot be fully characterised in our study, due to the lacking data of ventilation parameters. Although the IMV rates between patients included in the subgroup analysis and the overall groups remained similar (due to matching), it may be expected that ventilation was more invasive in patients with respiratory disease due to COVID-19, leading to the aforementioned effects.

### COVID-19 associated risk for RRT

Despite propensity score matching accounting for differences in disease severity, IMV, age and sex, the risk for RRT requirement was significantly higher in non-COVID-19 patients during the first and second day after ICU admission, but this changed with duration of ICU stay. Similar results were found in another analysis comparing two ARDS cohorts (with and without COVID-19): in the first 48 h after initiation of IMV the AKI rate was significantly lower in the COVID-19 group, while this trend reversed with ongoing ICU stay [32]. However, our sensitivity analysis restricted to patients with respiratory disease no crossing Kaplan-Meier curves, but generally higher risks for the initiation of RRT from day one were seen in the COVID-19 group. This, however, was limited to patients receiving IMV and no difference was found in patients treated with non-invasive respiratory support measures. In addition to already mentioned interactions between the lungs and the kidneys, which seem to become more relevant over time, presumably other factors contributed to the higher risk of COVID-19 patients over time. Secondary bacterial infections or ventilator-associated pneumonia become increasingly relevant at later stages of ICU stay [33]. Regardless of whether the secondary co-infections are bacterial, viral or fungal, patients have a higher risk of sepsis-associated AKI when ICU stay is prolonged [34]. Studies focusing on septic patients have reported AKI rates of more than 50% [35, 36]. Although the pathophysiology is not fully clarified yet, a multifactorial cause of sepsis-associated AKI is assumed, which shares many common features with COVID-19 associated AKI [37]. Additionally, during the pandemic, time related changes in the rate of RRT were reported in the literature: Lumlertgul et al. showed decreasing rates of RRT as the pandemic progressed, which was assumed to be associated with lower rates of IMV and lower PEEP [38]. Similar findings were reported by Mayerhöfer et al. [23]. In our cohort, no relevant changes in the rate of RRT could be observed during the selected observation period (2020–2022) with the exception of a singular spike in Q2 of 2020, which would correlate with the first wave of COVID-19 reported in the aforementioned studies [38]. However, since our analysis considered IMV in the propensity score matching it seems unlikely that changes in respiratory support measures affected the findings of our study.

### RRT associated mortality

Survival probability was significantly lower for COVID-19 compared to non-COVID-19 patients with RRT. This observation is in accordance with the results from other studies [32, 39] investigating AKI. Since in both groups most of the patients required IMV from the first day of ICU stay and the median delay to RRT initiation was 5 days, AKI mostly occurred after ARDS in our analysis. Studies showed that the occurrence of ARDS with AKI was associated with worse outcome [40, 41]. In our study, beside age and high SOFA score especially IMV concurrent with RRT was a risk factor for ICU death in patients receiving RRT. The requirement of RRT categorizes AKI as Kidney Disease: Improving Global Outcomes (KDIGO) stage 3 for which a previously published analysis showed a twofold higher mortality rate than expected by SAPS 3 [7]. This correlates well to our findings. There are multiple reasons that might explain these findings. In the COVID-19 group the occurrence of AKI could have led to higher mortality, as previous analyses have already shown increased mortality in sepsis-acquired AKI compared to non-septic AKI [42]. In the literature, higher mortality rates in COVID-19 patients are assumed to be caused by limited resources and logistic considerations during the pandemic [43]. However, we compared non-COVID-19 and COVID-19 patients treated during the same periods in the same country and limitation of resources should have impacted both groups alike. Finally, initiation or decision of withholding RRT treatment may have varied between COVID-19 and non-COVID-19 patients. To address this potential factor, we performed an additional analysis of RRT and death as combined endpoint, which showed the same pattern as for RRT onlyreducing the likelihood of any treatment bias.

### Strengths and limitations

The main strength of this study is the use of a large, representative real-world database including more than 150 Austrian ICUs.

Several limitations of this study must be mentioned. First, its observational design restricts causal interferences and may lead to bias. We sought to mitigate the bias by including potential confounding factors in the statistical models and performing propensity score matching to support meaningful comparisons. Secondly, several parameters such as KDIGO stage, medication (e.g. diuretics) or specific ventilator settings were not available from the database. Third, the overall groups (COVID-19 versus non-COVID) differed in the admission diagnosis respiratory failure. To compensate for this imbalance, we did a sensitivity analysis which restricted to patients with admission diagnosis of a respiratory disease which confirmed our results. Fourth, criteria for initiation or withholding RRT were not pre-specified, leaving the decision to perform RRT to the clinicians and, thus, some regional differences in treatment patterns cannot be completely excluded.

## Conclusion

The analysis evaluated that IMV as well as COVID-19 are associated with elevated risk for initiation of RRT. The association between IMV and increased risk of RRT initiation was given for all investigated time intervals. Additionally, COVID-19 patients showed an increased risk for RRT initiation during the entire ICU stay within patients admitted to an ICU due to respiratory disease. In COVID-19 patients treated with RRT, the risk of death was significantly higher compared to non-COVID-19 patients.

## Electronic supplementary material

Below is the link to the electronic supplementary material.

Supplementary Material 1

## Data Availability

The datasets generated and/or analysed during the current study are not publicly available but are available from the corresponding author on reasonable request.
