# Supplementary material for: Impact of mechanical ventilation on severe acute kidney injury in critically ill patients with and without COVID-19 – a multicentre propensity matched analysis
Source: Ann Intensive Care. 2025 Jan 25;15:17. doi: 10.1186/s13613-025-01424-4 (PMC11762028; doi:10.1186/s13613-025-01424-4)
Supplement: Supplementary file 1 — Supplementary Material 1 [file 13613_2025_1424_MOESM1_ESM.docx]

Electronic supplemental material

**Impact of mechanical ventilation on Severe Acute Kidney Injury in critically ill patients with and without COVID-19 – a multicentre propensity matched analysis**

Fabian Perschinka^1^, Timo Mayerhöfer^1^, Teresa Engelbrecht^2^, Alexandra Graf^2^, Paul Zajic^3^, Philipp Metnitz^3^, Michael Joannidis^1^

Table of contents

[ESM Table 1: Classification of diseases into different main diagnoses 3](#_Toc183117345)

[ESM Table 2: Used R packages 5](#_Toc183117346)

[ESM Table 3: Baseline characteristics of the unmatched cohorts 6](#_Toc183117347)

[ESM Table 4: Cox proportional hazards for RRT requirement 8](#_Toc183117348)

[ESM Table 5: Cox proportional hazards for RRT initiation in the cohorts additionally matched with the covariate ‘body mass index (BMI)’ 9](#_Toc183117349)

[ESM Table 6: Cox proportional hazards for RRT initiation in the cohorts matched with each comorbidity of the SAPS 3 instead of the SAPS 3 points 10](#_Toc183117350)

[ESM Table 7: Cox proportional hazards for RRT requirement or ICU death 11](#_Toc183117351)

[ESM Table 8: Baseline characteristics restricted to patients admitted to the ICU due to respiratory disease 12](#_Toc183117352)

[ESM Table 9: Hazard ratio for RRT initiation restricted to COVID-19 patients admitted to the ICU due to respiratory disease 13](#_Toc183117353)

[ESM Table 10: Cox proportional hazards for RRT initiation in the cohorts additionally matched with the covariate “BMI” restricted to patients admitted to the ICU due to respiratory disease 14](#_Toc183117354)

[ESM Figure 1: Enrolment flowchart 15](#_Toc183117355)

[ESM Figure 2: RRT rates per quarter in the years of the observational period 16](#_Toc183117356)

[ESM Figure 3: Hazard ratio for RRT initiation or ICU death 17](#_Toc183117357)

[ESM Figure 4: Kaplan-Meier curve of time to RRT or ICU-death overall and divided in age groups 18](#_Toc183117358)

[ESM Figure 5: Kaplan-Meier curve for ICU mortality and hospital mortality after RRT initiation 19](#_Toc183117359)

[ESM Figure 6: Comparison of time to RRT of patients assigned to the IMV group and patients in the non-IMV group restricted to patients admitted to the ICU due to respiratory disease 20](#_Toc183117360)

[ESM Figure 7: Kaplan-Meier curve for ICU mortality and hospital mortality after RRT initiation restricted to patients ICU-admitted due to respiratory disease 21](#_Toc183117361)

# ESM Table 1: Classification of diseases into different main diagnoses

| Metabolic Disease | Metabolic coma |
| --- | --- |
|  | Drug intoxication |
|  | Other intoxication |
|  | Endocrinopathy |
|  | Diabetic ketoacidosis |
|  | Other metabolic disease |
| Respiratory Disease | ALI/ARDS |
|  | Pneumonia |
|  | Acute respiratory insufficiency in COPD |
|  | Asthma bronchiale |
|  | Pulmonary embolism |
|  | Airway obstruction |
|  | Pneumothorax |
|  | Pleural effusion |
|  | Hemoptysis |
|  | Neoplasm |
|  | Pulmonary edema |
|  | Aspiration |
|  | Other respiratory disease |
| Cardiovascular disease | Asystole |
|  | Myocardial infarction |
|  | Myocarditis |
|  | Unstable angina |
|  | Heart failure |
|  | Pericardial effusion |
|  | Pulmonary edema (cardiac) |
|  | Hypertensive crisis |
|  | Arrhythmia |
|  | Cardioversion |
|  | Aortic aneurysm |
|  | Other cardiovascular disease |
| Shock | Hypovolemic shock |
|  | Septic shock |
|  | Cardiogenic shock |
|  | Anaphylactic shock |
|  | Mixed shock |
|  | Shock of unknown cause |
| Renal disease | Acute kidney failure |
|  | Chronic kidney disease |
|  | Crush syndrome |
|  | Other cause for renal failure |
| Neurological disease | Cerebrovascular insult |
|  | Intracranial masses |
|  | Meningitis/Encephalitis |
|  | Epileptic seizure |
|  | Myopathy/myasthenia |
|  | Degenerative disease |
|  | Coma, Stupor |
|  | Focal neurological deficit |
|  | Other neurological disease |
| Sepsis | Sepsis, suspicion |
|  | Sepsis, documented |
|  | Sepsis, undocumented |
|  | Sepsis, unknown |
| Trauma (not operated) | Head/spinal injury |
|  | Thoracic trauma |
|  | Abdominal trauma |
|  | Skeletal trauma |
|  | Polytrauma |
|  | Burn |
|  | Other trauma |
| Gastrointestinal disease | Liver failure |
|  | Pancreatitis |
|  | Upper gastrointestinal bleeding |
|  | Lower gastrointestinal bleeding |
|  | Acute abdomen |
|  | Other gastrointestinal disease |
| Hematological disease | Bone marrow transplant |
|  | Disseminated intravascular coagulation |
|  | Malignant hematological disease |
|  | Non-malignant hematological disease |
| Other | Other non-operative reason for admission |
|  | Preeclampsia/eclampsia |
|  | HELLP syndrome |
|  | Pregnancy |

# ESM Table 2: Used R packages

| *Packages* | *Version* |
| --- | --- |
| *scales* | *1.3.0* |
| *dplyr* | *1.1.4* |
| *stats* | *4.4.1* |
| *tibble* | *3.2.1* |
| *tidyr* | *1.3.1* |
| *ggpubr* | *0.6.0* |
| *gtsummary* | *1.7.2* |
| *base* | *4.4.1* |
| *flextable* | *0.9.6* |
| *forecast* | *1.0.0* |
| *ggplot2* | *3.5.1* |
| *stringr* | *1.5.1* |
| *survival* | *3.7.0* |
| *suryminer* | *0.4.9* |
| *Matchlt* | *4.5.0* |
| *lubridate* | *1.9.3* |
| *purrr* | *1.0.2* |
| *utils* | *4.4.1* |

# ESM Table 3: Baseline characteristics of the unmatched cohorts

|  | Overall  (n = 28572) | Non-COVID-19  (n = 23991) | COVID-19  (n = 4581) |
| --- | --- | --- | --- |
|  |  |  |  |
| Age | 68.0 (58.0 – 77.0) | 69.0 (58.0 – 78.0) | 66.0 (56.0 – 74.0) |
| Sex |  |  |  |
| Female | 10691 (37.4%) | 9275 (38.7%) | 1416 (30.9%) |
| Male | 17880 (62.6%) | 14715 (61.3%) | 3165 (69.1%) |
| SAPS 3 | 55.0 (47.0 – 65.0) | 56.0 (47.0 – 66.0) | 54.0 (47.0 – 62.0) |
|  |  |  |  |
| **Main diagnosis** |  |  |  |
| Metabolic disease | 827 (2.9%) | 805 (3.4%) | 22 (0.5%) |
| Respiratory disease | 9851 (34.5%) | 6154 (25.7%) | 3697 (80.7%) |
| Cardiovascular disease | 4458 (15.6%) | 4390 (18.3%) | 68 (1.5%) |
| Shock | 995 (3.5%) | 976 (4.1%) | 19 (0.4%) |
| Renal disease | 1274 (4.5% | 1220 (5.1%) | 54 (1.2%) |
| Neurologic disease | 2128 (7.4%) | 2067 (8.6%) | 61 (1.3%) |
| Sepsis | 867 (3.0%) | 844 (3.5%) | 23 (0.5%) |
| Trauma (not operated) | 1375 (4.8%) | 1351 (5.6%) | 24 (0.5%) |
| Gastrointestinal disease | 1003 (3.5%) | 992 (4.1%) | 11 (0.2%) |
| Hematologic disease | 102 (0.4%) | 99 (0.4%) | 3 (0.1%) |
| Medical | 390 (1.4%) | 349 (1.5% | 41 (0.9%) |
| Other | 5302 (18.6%) | 4744 (19.8%) | 558 (12.2%) |
|  |  |  |  |
|  |  |  |  |
| **Length of stay and mortality** |  |  |  |
| ICU LOS | 6.0 (4.0 – 12.0) | 5.0 (4.0 – 10.0) | 11.0 (6.0 – 19.0) |
| Hospital LOS | 16.0 (9.0 – 28.0) | 15.0 (8.0 – 27.0) | 20.0 (13.0 – 32.0) |
| ICU mortality | 4962 (17.4%) | 3624 (15.1%) | 1338 (29.2%) |
| Hospital mortality | 7196 (25.5%) | 5625 (23.8%) | 1571 (34.6%) |
| SAPS Observed/Expected mortality ratio | 0.80 (0.79 – 0.82) | 0.73 (0.72 – 0.75) | 1.22 (1.18 – 1.26) |
|  |  |  |  |
| **Most invasive airway (before RRT)** |  |  |  |
| No support | 734 (2.6%) | 703 (2.9%) | 31 (0.7%) |
| O2 (mask or nasal cannula) | 7318 (25.6%) | 7118 (29.7%) | 200 (4.4%) |
| NHFO | 469 (1.6%) | 292 (1.2%) | 177 (3.9%) |
| NIV (mask or helmet) | 6314 (22.1%) | 4987 (20.8%) | 1327 (29.0%) |
| Endotracheal tube | 10169 (35.6%) | 7967 (33.2%) | 2202 (48.1%) |
| Tracheal cannula | 2430 (8.5%) | 1846 (7.7%) | 584 (12.7%) |
| Missing | 21 (0.1%) | 21 (0.1%) | 0 |
|  |  |  |  |
| **Most invasive ventilation mode (before RRT)** |  |  |  |
| Spontaneous breathing | 8846 (31.0%) | 8418 (35.1%) | 428 (9.3%) |
| CPAP | 4258 (14.9%) | 3439 (14.3%) | 819 (17.9%) |
| Assisted | 2909 (10.2%) | 2257 (9.4%) | 652 (14.2%) |
| BIPAP | 4377 (15.3%) | 3492 (14.6%) | 885 (19.3%) |
| HF-Ventilation | 84 (0.3%) | 37 (0.2%) | 47 (1.0%) |
| Controlled | 6922 (24.2%) | 5243 (21.9%) | 1679 (36.7%) |
| Controlled & HF-Ventilation | 31 (0.1%) | 23 (0.1%) | 8 (0.2%) |
| Missing | 28 (0.1%) | 25 (0.1%) | 3 (0.1%) |
|  |  |  |  |
| **Differences in initiation of RRT and IMV** |  |  |  |
| RRT performed | 3899 (13.6%) | 3319 (13.8%) | 580 (12.7%) |
| RRT and IMV simultaneously | 2203 (7.7%) | 1692 (7.1%) | 511 (11.2%) |
| RRT initiation after IMV | 1445 (5.1%) | 1036 (4.3%) | 409 (8.9%) |
| Initiation of RRT and IMV on the same day | 448 (1.6%) | 394 (1.6%) | 54 (1.2%) |
| RRT initiation before IMV | 310 (1.1%) | 262 (1.1%) | 48 (1.1%) |
| RRT initiation after IMV (days) | 2.0 (1.0 – 7.0) | 2.0 (1.0 – 5.0) | 5.0 (2.0 – 11.0) |
| RRT initiation before IMV (days) | 2.0 (1.0 – 6.0) | 2.0 (1.0 – 6.0) | 2.0 (1.0 – 4.0) |
| Sum of the days of RRT | 5.0 (3.0 – 9.0) | 4.0 (3.0 – 8.0) | 6.0 (3.0 – 13.0) |
| Sum of the days of IMV | 0.0 (0.0 – 5.0) | 0.0 (0.0 – 4.0) | 4.0 (0.0 – 12.0) |

Legend: SAPS 3 - Simplified acute physiology score 3; ICU LOS – Intensive care unit length of stay; Hospital LOS - Hospital length of stay; NIV – Non-invasive ventilation; CPAP – Continuous positive airway pressure; NHFO – Nasal high-flow oxygen; BIPAP - Biphasic positive airway pressure; HF-Ventilation – High-frequency ventilation; IMV – Invasive mechanical ventilation; RRT – Renal replacement therapy.

# ESM Table 4: Cox proportional hazards for RRT requirement

| 1. **Restricted to non-COVID-19 patients** | | **HR** | **lower** | **upper** | **p-value** |
| --- | --- | --- | --- | --- | --- |
| **IMV: Yes vs. No** | |  |  |  |  |
|  | Days 1 to 7 | 0.94 | 0.72 | 1.25 | 0.687 |
|  | Days 8 to 14 | 1.07 | 0.52 | 2.19 | 0.862 |
|  | Days >= 15 | 1.88 | 0.45 | 7.81 | 0.387 |
| **Age: Reference: 18-59** | |  |  |  |  |
|  | 60-69 | 1.02 | 0.83 | 1.26 | 0.841 |
|  | 70-79 | 0.98 | 0.77 | 1.24 | 0.859 |
|  | 80-96 | 0.66 | 0.47 | 0.92 | **0.015** |
| **SAPS 3 score: Reference: Low** | |  |  |  |  |
|  | Medium | 1.37 | 1.02 | 1.85 | **0.036** |
|  | High | 2.67 | 2.03 | 3.51 | **< 0.0001** |
|  |  |  |  |  |  |
| **Sex: Male vs. Female** |  | 1.28 | 1.08 | 1.51 | **0.005** |
|  |  |  |  |  |  |
| 1. **Restricted to COVID-19 patients** | | **HR** | **lower** | **upper** | **p-value** |
| **IMV: Yes vs. No** | |  |  |  |  |
|  | Days 1 to 7 | 2.35 | 1.81 | 3.06 | **< 0.0001** |
|  | Days 8 to 14 | 2.41 | 1.53 | 3.81 | **< 0.0001** |
|  | Days >= 15 | 3.42 | 1.71 | 6.84 | **0.001** |
| **Age: Reference: 18-59** | |  |  |  |  |
|  | 60-69 | 1.03 | 0.77 | 1.39 | 0.825 |
|  | 70-79 | 0.97 | 0.73 | 1.29 | 0.834 |
|  | 80-96 | 0.55 | 0.36 | 0.84 | **0.006** |
| **SAPS 3 score: Reference: Low** | |  |  |  |  |
|  | Medium | 1.55 | 1.15 | 2.10 | **0.004** |
|  | High | 3.31 | 2.41 | 4.54 | **< 0.0001** |
|  |  |  |  |  |  |
| **Sex: Male vs. Female** |  | 1.43 | 1.17 | 1.75 | **< 0.0001** |

Legend: HR – Hazard ratio; IMV – invasive mechanical ventilation; RRT – renal replacement therapy; SAPS 3 - Simplified Acute Physiology Score 3.

# ESM Table 5: Cox proportional hazards for RRT initiation in the cohorts additionally matched with the covariate ‘body mass index (BMI)’

|  |  | **HR** | **Lower** | **upper** | **p-value** |
| --- | --- | --- | --- | --- | --- |
| **COVID-19 vs. Control** |  |  |  |  |  |
|  | Day 1 | 0.49 | 0.26 | 0.93 | **0.030** |
|  | Day 2 | 0.70 | 0.45 | 1.09 | 0.115 |
|  | Days 3 to 7 | 1.11 | 0.79 | 1.55 | 0.540 |
|  | Days 8 to 14 | 1.79 | 1.19 | 2.70 | **0.006** |
|  | Days 15 to 21 | 1.50 | 0.84 | 2.68 | 0.170 |
|  | Days >= 21 | 4.25 | 1.78 | 10.17 | **0.001** |
| **IMV: Yes vs. No** |  |  |  |  |  |
|  | Days 1 to 7 | 1.33 | 1.04 | 1.70 | **0.025** |
|  | Days 8 to 14 | 1.66 | 1.06 | 2.58 | **0.026** |
|  | Days >= 15 | 1.70 | 0.81 | 3.53 | 0.158 |
| **Age: Reference: 18-59** |  |  |  |  |  |
|  | 60-69 | 0.89 | 0.73 | 1.08 | 0.241 |
|  | 70-79 | 0.90 | 0.72 | 1.12 | 0.334 |
|  | 80-95 | 0.50 | 0.36 | 0.69 | **< 0.0001** |
| **Body mass index (BMI): Reference Normal weight** |  |  |  |  |  |
|  | <18.5 | 0.78 | 0.35 | 1.72 | 0.533 |
|  | 25 – 29.9 | 0.99 | 0.78 | 1.24 | 0.898 |
|  | >30 | 1.20 | 0.96 | 1.49 | 0.110 |
| **SAPS 3 score: Reference: Low** |  |  |  |  |  |
|  | Medium | 1.67 | 1.25 | 2.24 | **0.001** |
|  | High | 3.34 | 2.43 | 4.60 | **< 0.0001** |
| **Sex: Male vs. Female** |  | 1.40 | 1.16 | 1.68 | **< 0.0001** |

Legend: HR – Hazard ratio; IMV – invasive mechanical ventilation; RRT – renal replacement therapy; SAPS 3 - Simplified Acute Physiology Score 3.

Normal weight: BMI 18.5 – 24.9.

# ESM Table 6: Cox proportional hazards for RRT initiation in the cohorts matched with each comorbidity of the SAPS 3 instead of the SAPS 3 points

|  |  | **HR** | **Lower** | **upper** | **p-value** |
| --- | --- | --- | --- | --- | --- |
| **COVID-19 vs. Control** |  |  |  |  |  |
|  | Day 1 | 0.26 | 0.15 | 0.44 | **< 0.0001** |
|  | Day 2 | 0.51 | 0.33 | 0.80 | **0.003** |
|  | Days 3 to 7 | 0.90 | 0.68 | 1.17 | 0.423 |
|  | Days 8 to 14 | 1.30 | 0.92 | 1.84 | 0.141 |
|  | Days 15 to 21 | 2.08 | 1.14 | 3.78 | **0.017** |
|  | Days >= 21 | 2.71 | 1.45 | 5.05 | **0.002** |
| **IMV: Yes vs. No** |  |  |  |  |  |
|  | Days 1 to 7 | 1.78 | 1.41 | 2.25 | **< 0.0001** |
|  | Days 8 to 14 | 2.33 | 1.58 | 3.41 | **< 0.0001** |
|  | Days >= 15 | 3.42 | 1.77 | 6.60 | **< 0.0001** |
| **Age: Reference: 18-59** |  |  |  |  |  |
|  | 60-69 | 1.21 | 1.04 | 1.40 | **0.014** |
|  | 70-79 | 1.29 | 1.07 | 1.54 | **0.007** |
|  | 80-95 | 0.99 | 0.76 | 1.28 | 0.922 |
|  |  |  |  |  |  |
| **Cancer therapy: Yes vs No** |  | 1.33 | 1.06 | 1.68 | **0.016** |
| **Chronic HF (NYHA IV): Yes vs No** |  | 1.95 | 1.15 | 3.33 | **0.014** |
| **Cirrhosis: Yes vs No** |  | 1.71 | 1.15 | 2.54 | **0.008** |
| **Haematological cancer: Yes vs No** |  | 1.27 | 0.90 | 1.79 | 0.166 |
| **Metastatic cancer: Yes vs No** |  | 0.73 | 0.44 | 1.18 | 0.199 |
|  |  |  |  |  |  |
| **Sex: Male vs. Female** |  | 1.29 | 1.14 | 1.47 | **< 0.0001** |

Legend: HR – Hazard ratio; IMV – invasive mechanical ventilation; RRT – renal replacement therapy; HF – heart failure; NYHA - New York Heart Association.

# ESM Table 7: Cox proportional hazards for RRT requirement or ICU death

|  |  | **HR** | **lower** | **upper** | **p-value** |
| --- | --- | --- | --- | --- | --- |
| **COVID-19 vs. Control** | |  |  |  |  |
|  | Day 1 | 0.43 | 0.27 | 0.68 | **< 0.0001** |
|  | Day 2 | 0.60 | 0.39 | 0.92 | **0.018** |
|  | Days 3 to 7 | 1.01 | 0.85 | 1.20 | 0.917 |
|  | Days 8 to 14 | 1.30 | 1.04 | 1.63 | **0.019** |
|  | Days 15 to 21 | 1.59 | 1.22 | 2.07 | **0.001** |
|  | Days >= 21 | 1.89 | 1.40 | 2.55 | **< 0.0001** |
| **IMV: Yes vs. No** | |  |  |  |  |
|  | Days 1 to 7 | 1.25 | 1.10 | 1.42 | **0.001** |
|  | Days 8 to 14 | 1.45 | 1.21 | 1.74 | **< 0.0001** |
|  | Days >= 15 | 1.82 | 1.41 | 2.35 | **< 0.0001** |
| **Age: Reference: 18-59** | |  |  |  |  |
|  | 60-69 | 1.32 | 1.15 | 1.51 | **< 0.0001** |
|  | 70-79 | 1.53 | 1.32 | 1.77 | **< 0.0001** |
|  | 80-96 | 1.82 | 1.51 | 2.19 | **< 0.0001** |
| **SAPS 3 score: Reference: Low** | |  |  |  |  |
|  | Medium | 1.50 | 1.29 | 1.73 | **< 0.0001** |
|  | High | 2.35 | 2.00 | 2.75 | **< 0.0001** |
|  |  |  |  |  |  |
| **Sex: Male vs. Female** |  | 1.17 | 1.08 | 1.28 | **< 0.0001** |

Legend: HR – Hazard ratio; IMV – invasive mechanical ventilation; RRT – renal replacement therapy; SAPS 3 - Simplified Acute Physiology Score 3.

# ESM Table 8: Baseline characteristics restricted to patients admitted to the ICU due to respiratory disease

|  | Overall  (n = 5474) | Non-COVID-19  (n = 2737) | COVID-19  (n = 2737) |
| --- | --- | --- | --- |
|  |  |  |  |
| Age | 67.0 (58.0 – 75.0) | 67.0 (58.0 – 75.0) | 67.0 (57.0 – 74.0) |
| Sex |  |  |  |
| Female | 1823 (33.3%) | 857 (31.3%) | 966 (35.3%) |
| Male | 3651 (66.7%) | 1880 (68.7%) | 1771 (64.7%) |
| SAPS 3 | 54.0 (47.0 – 62.0) | 54.0 (47.0 – 62.0) | 54.0 (47.0 – 61.0) |
|  |  |  |  |
| **Length of stay and mortality** |  |  |  |
| ICU LOS | 9.0 (5.0 – 16.0) | 7.0 (4.0 – 13.0) | 10.0 (6.0 – 18.0) |
| Hospital LOS | 19.0 (11.0 – 30.0) | 17.0 (10.0 – 29.0) | 20.0 (13.0 – 31.0) |
| ICU mortality | 1315 (24.0%) | 506 (18.5%) | 809 (29.6%) |
| Hospital mortality | 1686 (31.0%) | 746 (27.4%) | 940 (34.6%) |
| SAPS Observed/Expected mortality ratio | 1.11 (1.07 – 1.15) | 0.97 (0.92 – 1.03) | 1.25 (1.20 – 1.31) |
|  |  |  |  |
| **Patients who ever received BEFORE RRT...** |  |  |  |
| **Most invasive airway** |  |  |  |
| No support | 23 (0.4%) | 11 (0.4%) | 12 (0.4%) |
| O2 (mask or nasal cannula) | 362 (6.6%) | 271 (9.9%) | 91 (3.3%) |
| NHFO | 165 (3.0%) | 47 (1.7%) | 118 (4.3%) |
| NIV (mask or helmet) | 1726 (31.5%) | 811 (29.6%) | 915 (33.4%) |
| Endotracheal tube | 2396 (43.8%) | 1150 (42.0%) | 1246 (45.5%) |
| Tracheal cannula | 768 (14.0%) | 432 (15.8%) | 336 (12.3%) |
| Missing | 2 (0.0%) | 2 (0.1%) | 0 (0.0%) |
|  |  |  |  |
| **Most invasive ventilation mode** |  |  |  |
| Spontaneous breathing | 590 (10.8%) | 357 (13.0%) | 233 (8.5%) |
| CPAP | 1028 (18.8%) | 452 (16.5%) | 576 (21.0%) |
| Assisted | 963 (17.6%) | 524 (19.1%) | 439 (16.0%) |
| BIPAP | 1075 (19.6%) | 585 (21.4%) | 490 (17.9%) |
| HF-Ventilation | 38 (0.7%) | 11 (0.4%) | 27 (1.0%) |
| Controlled | 1738 (31.8%) | 792 (28.9%) | 946 (34.6%) |
| Controlled & HF-Ventilation | 7 (0.1%) | 1 (0.0%) | 6 (0.2%) |
| Missing | 3 (0.1%) | 2 (0.1%) | 1 (0.0%) |
|  |  |  |  |
| **Differences in initiation of RRT and IMV** |  |  |  |
| RRT performed | 515 (9.4%) | 206 (7.5%) | 309 (11.3%) |
| RRT and IMV simultaneously | 446 (8.2%) | 168 (6.1%) | 278 (10.2%) |
| RRT initiation after IMV | 373 (6.8%) | 144 (5.2%) | 229 (8.4%) |
| Initiation of RRT and IMV on the same day | 50 (0.9%) | 20 (0.7%) | 30 (1.1%) |
| RRT initiation before IMV | 23 (0.4%) | 4 (0.2%) | 19 (0.7%) |
| RRT initiation after IMV (days) | 4.0 (1.0 – 9.0) | 3.0 (1.0 – 6.5) | 5.0 (2.0 – 10.0) |
| RRT initiation before IMV (days) | 2.0 (1.0 – 4.0) | 2.5 (1.0 – 7.0) | 2.0 (1.0 – 3.5) |
| Sum of the days of RRT | 6.0 (3.0 – 12.0) | 6.0 (3.0 –11.0) | 6.0 (3.0 – 13.0) |
| Sum of the days of IMV | 2.0 (0.0 – 10.0) | 2.0 (0.0 – 7.0) | 4.0 (0.0 – 12.0) |

Legend: SAPS 3 - Simplified acute physiology score 3; ICU LOS – Intensive care unit length of stay; Hospital LOS - Hospital length of stay; NIV – Non-invasive ventilation; CPAP – Continuous positive airway pressure; NHFO – Nasal high-flow oxygen; BIPAP - Biphasic positive airway pressure; HF-Ventilation – High-frequency ventilation; IMV – Invasive mechanical ventilation; RRT – Renal replacement therapy.

# ESM Table 9: Hazard ratio for RRT initiation restricted to COVID-19 patients admitted to the ICU due to respiratory disease

|  |  | **HR** | **lower** | **upper** | **p-value** |
| --- | --- | --- | --- | --- | --- |
| **IMV: Yes vs. No** |  |  |  |  |  |
|  | Days 1 to 7 | 2.71 | 1.93 | 3.81 | **< 0.0001** |
|  | Days 8 to 14 | 3.05 | 1.79 | 5.20 | **< 0.0001** |
|  | Days >= 15 | 4.38 | 1.74 | 11.00 | **0.002** |
| **Age: Reference: 18-59** |  |  |  |  |  |
|  | 60-69 | 1.02 | 0.69 | 1.48 | 0.937 |
|  | 70-79 | 0.92 | 0.63 | 1.35 | 0.681 |
|  | 80-95 | 0.46 | 0.28 | 0.74 | **0.002** |
| **SAPS 3 score: Reference: Low** |  |  |  |  |  |
|  | Medium | 1.64 | 1.18 | 2.29 | **0.003** |
|  | High | 3.45 | 2.31 | 5.15 | **< 0.0001** |
| **Sex: Male vs. Female** |  | 1.51 | 1.20 | 1.89 | **< 0.0001** |

Legend: HR – Hazard ratio; IMV – invasive mechanical ventilation; RRT – renal replacement therapy; SAPS 3 - Simplified Acute Physiology Score 3.

# ESM Table 10: Cox proportional hazards for RRT initiation in the cohorts additionally matched with the covariate “BMI” restricted to patients admitted to the ICU due to respiratory disease

|  |  | **HR** | **Lower** | **upper** | **p-value** |
| --- | --- | --- | --- | --- | --- |
| **COVID-19 vs. Control** |  |  |  |  |  |
|  | Day 1 | 0.84 | 0.37 | 1.88 | 0.669 |
|  | Day 2 | 1.21 | 0.67 | 2.17 | 0.522 |
|  | Days 3 to 7 | 1.20 | 0.79 | 1.82 | 0.381 |
|  | Days 8 to 14 | 1.83 | 0.93 | 3.58 | 0.078 |
|  | Days 15 to 21 | 1.76 | 0.63 | 4.97 | 0.284 |
|  | Days >= 21 | 4.28 | 0.79 | 23.17 | 0.091 |
| **IMV: Yes vs. No** |  |  |  |  |  |
|  | Days 1 to 7 | 1.85 | 1.21 | 2.83 | **0.005** |
|  | Days 8 to 14 | 2.02 | 1.01 | 4.03 | **0.047** |
|  | Days >= 15 | 3.03 | 0.91 | 10.09 | 0.071 |
| **Age: Reference: 18-59** |  |  |  |  |  |
|  | 60-69 | 0.83 | 0.60 | 1.13 | 0.232 |
|  | 70-79 | 0.60 | 0.44 | 0.81 | **0.001** |
|  | 80-95 | 0.29 | 0.15 | 0.54 | **< 0.0001** |
| **Body mass index (BMI): Reference Normal weight** |  |  |  |  |  |
|  | <18.5 | 0.43 | 0.10 | 1.80 | 0.250 |
|  | 25 – 29.9 | 0.87 | 0.66 | 1.14 | 0.319 |
|  | >30 | 1.13 | 0.86 | 1.49 | 0.384 |
| **SAPS 3 score: Reference: Low** |  |  |  |  |  |
|  | Medium | 2.12 | 1.48 | 3.04 | **< 0.0001** |
|  | High | 4.32 | 2.95 | 6.32 | **< 0.0001** |
| **Sex: Male vs. Female** |  | 1.32 | 1.02 | 1.71 | **0.034** |

Legend: HR – Hazard ratio; IMV – invasive mechanical ventilation; RRT – renal replacement therapy; SAPS 3 - Simplified Acute Physiology Score 3.

Normal weight: BMI 18.5 – 24.9.

#
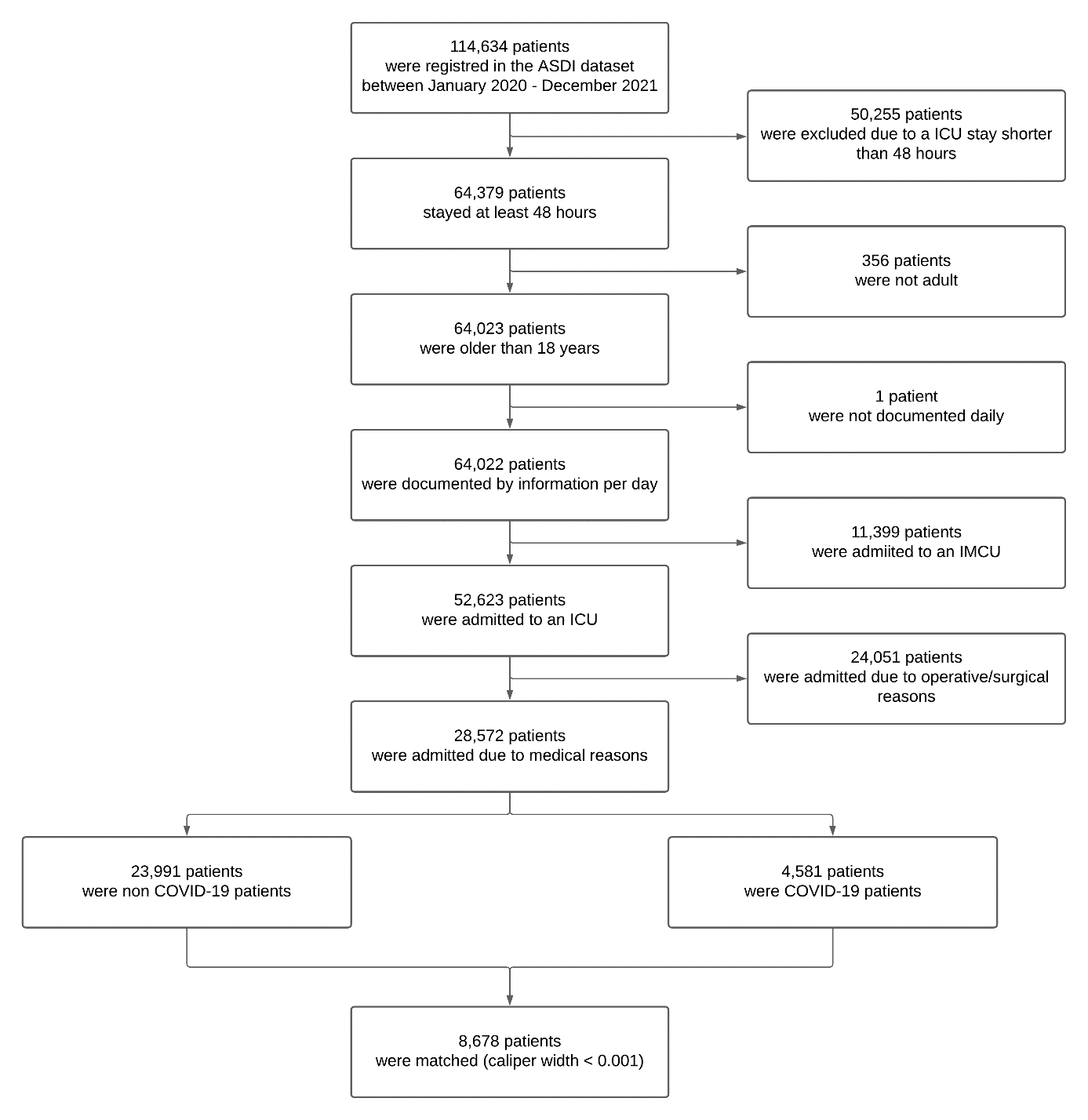
ESM Figure 1: Enrolment flowchart

Legend: ASDI - Austrian Centre for Documentation and Quality Assurance in Intensive Care; ICU – Intensive care unit; IMCU – Intermediate care unit.

#
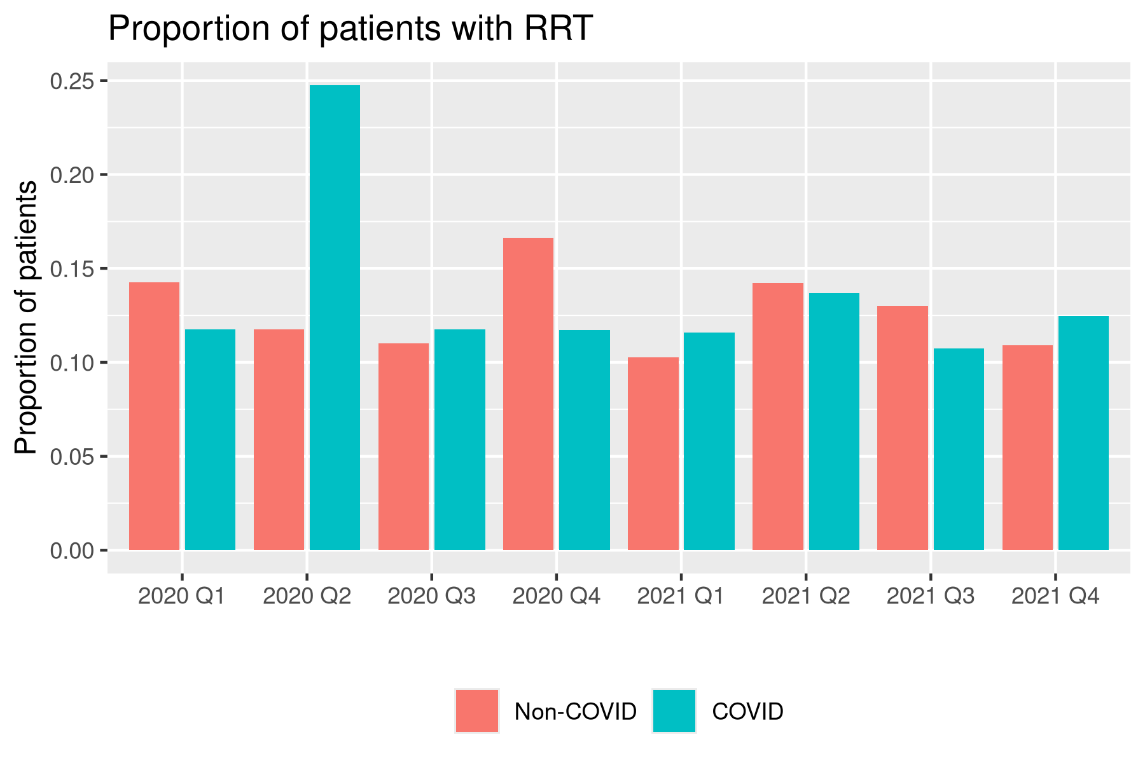
ESM Figure 2: RRT rates per quarter in the years of the observational period

Legend: RRT – renal replacement therapy; Q1 – January until March; Q2 – April until June; Q3 – July until September; Q4 – October until December

# ESM Figure 3: Hazard ratio for RRT initiation or ICU death


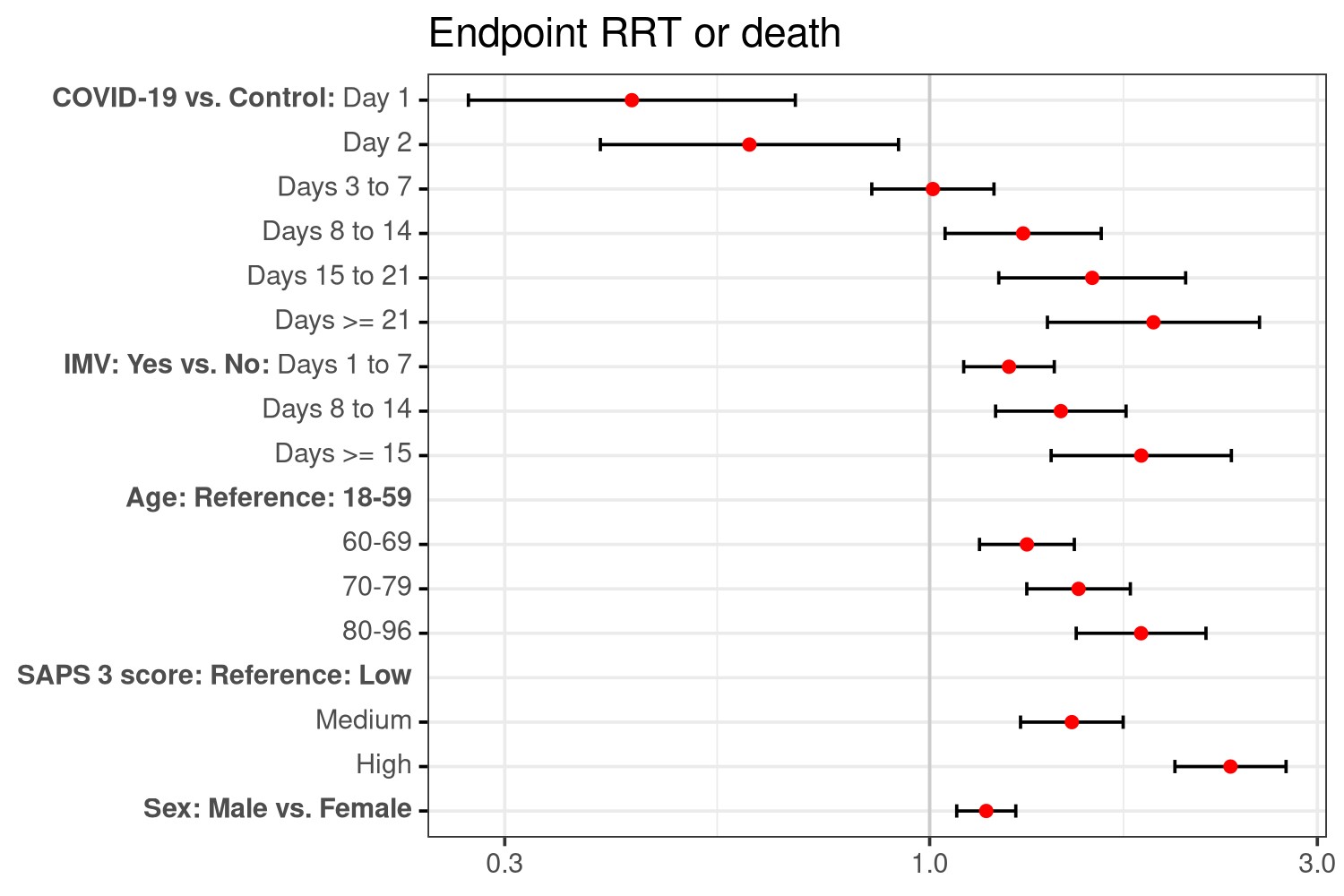
Legend: IMV: invasive mechanical ventilation.

# ESM Figure 4: Kaplan-Meier curve of time to RRT or ICU-death overall and divided in age groups

Legend: RRT- renal replacement therapy; ICU – intensive care unit.


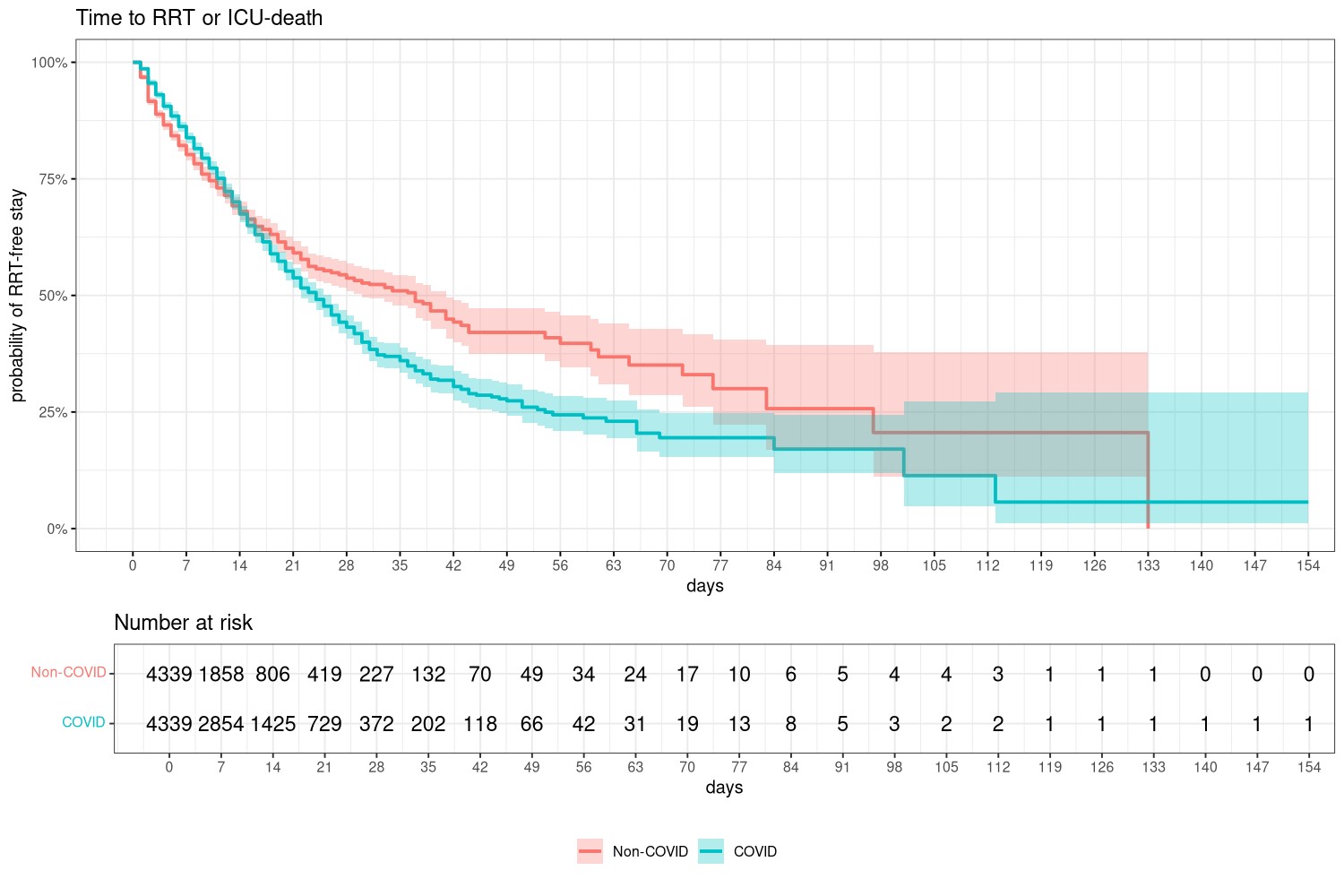

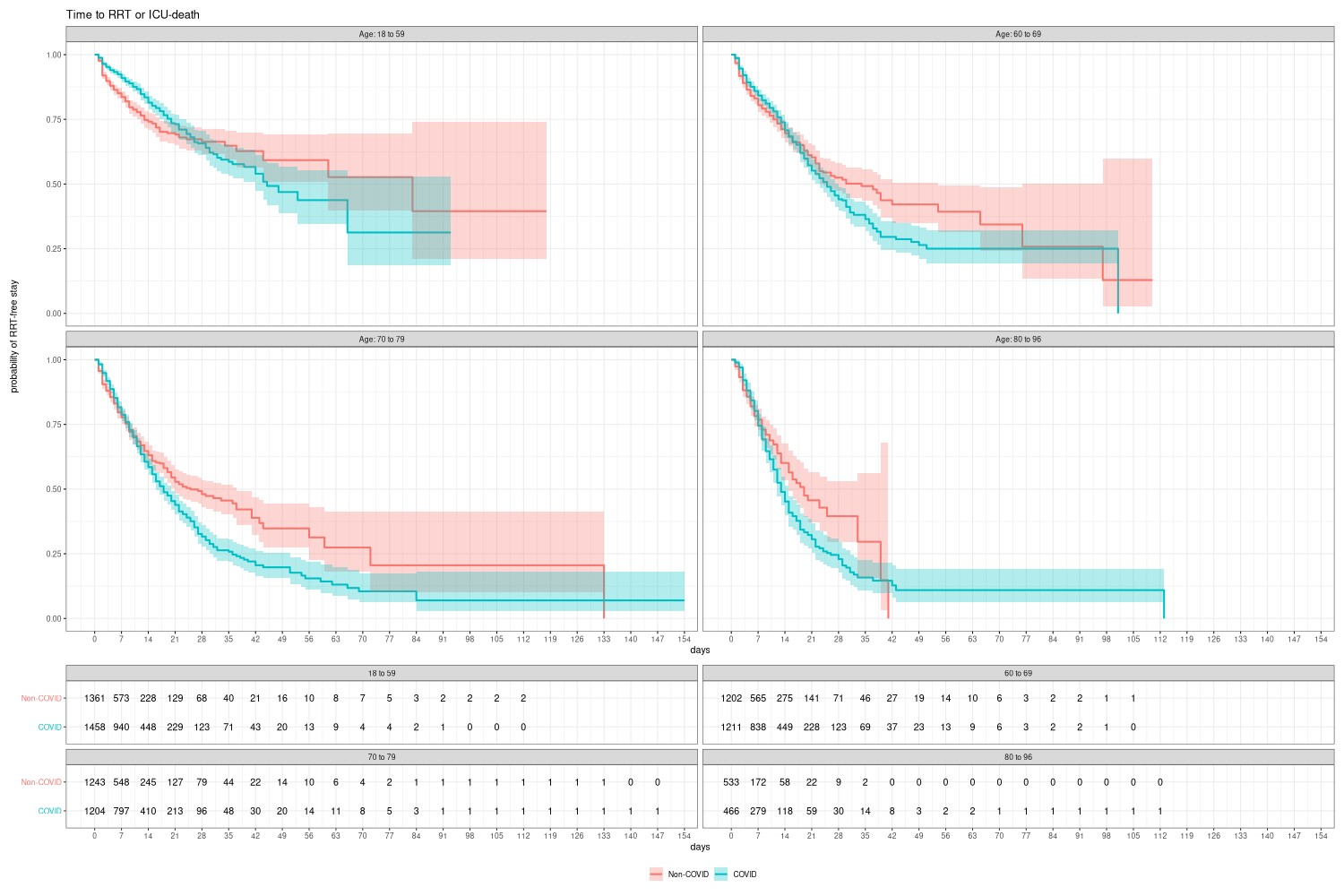

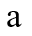

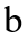


# ESM Figure 5: Kaplan-Meier curve for ICU mortality and hospital mortality after RRT initiation


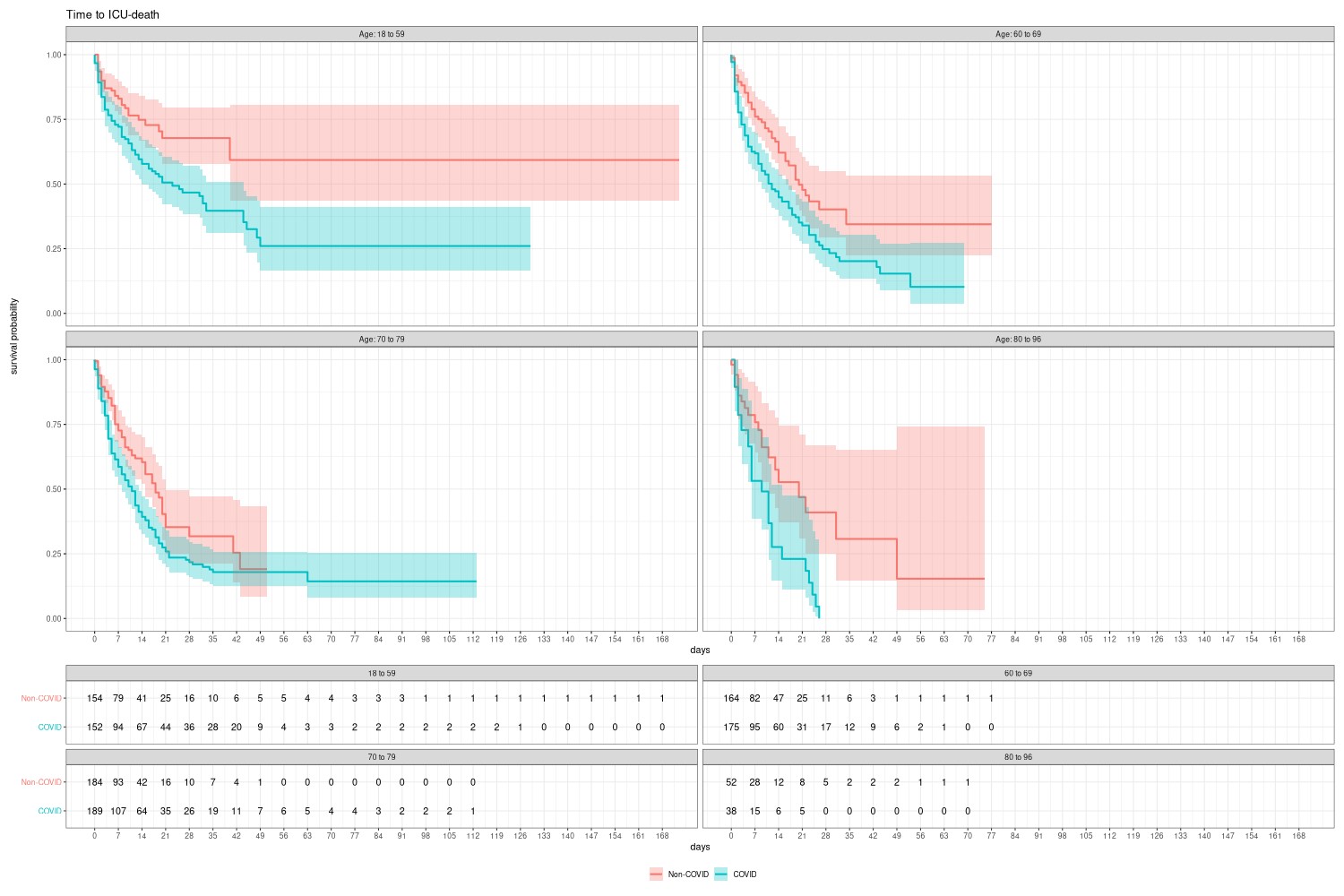

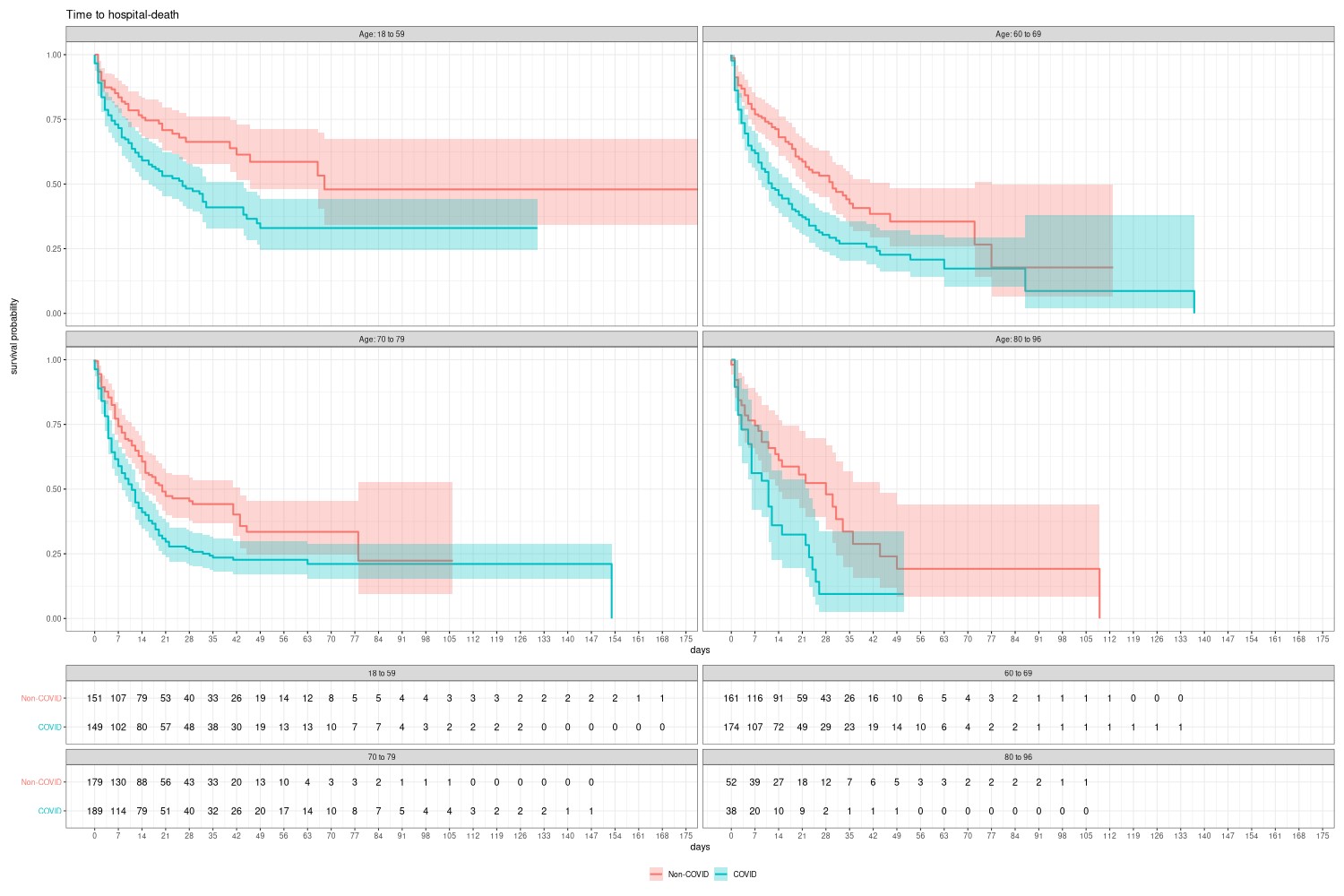

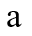

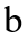


Legend: ICU – intensive care unit.

#
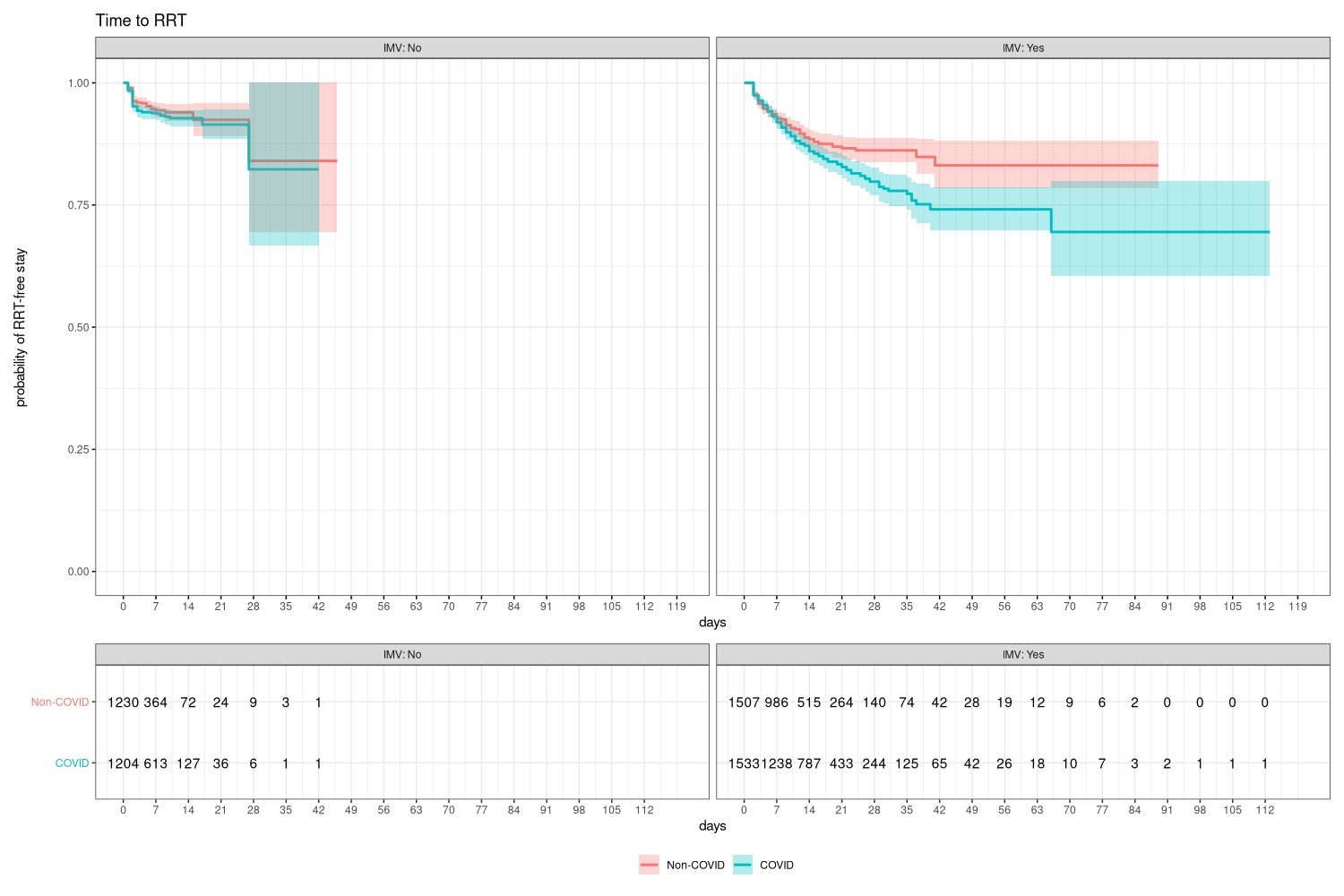
ESM Figure 6: Comparison of time to RRT of patients assigned to the IMV group and patients in the non-IMV group restricted to patients admitted to the ICU due to respiratory disease

Legend: IMV – invasive mechanical ventilation; RRT – Renal replacement therapy.

# ESM Figure 7: Kaplan-Meier curve for ICU mortality and hospital mortality after RRT initiation restricted to patients ICU-admitted due to respiratory disease


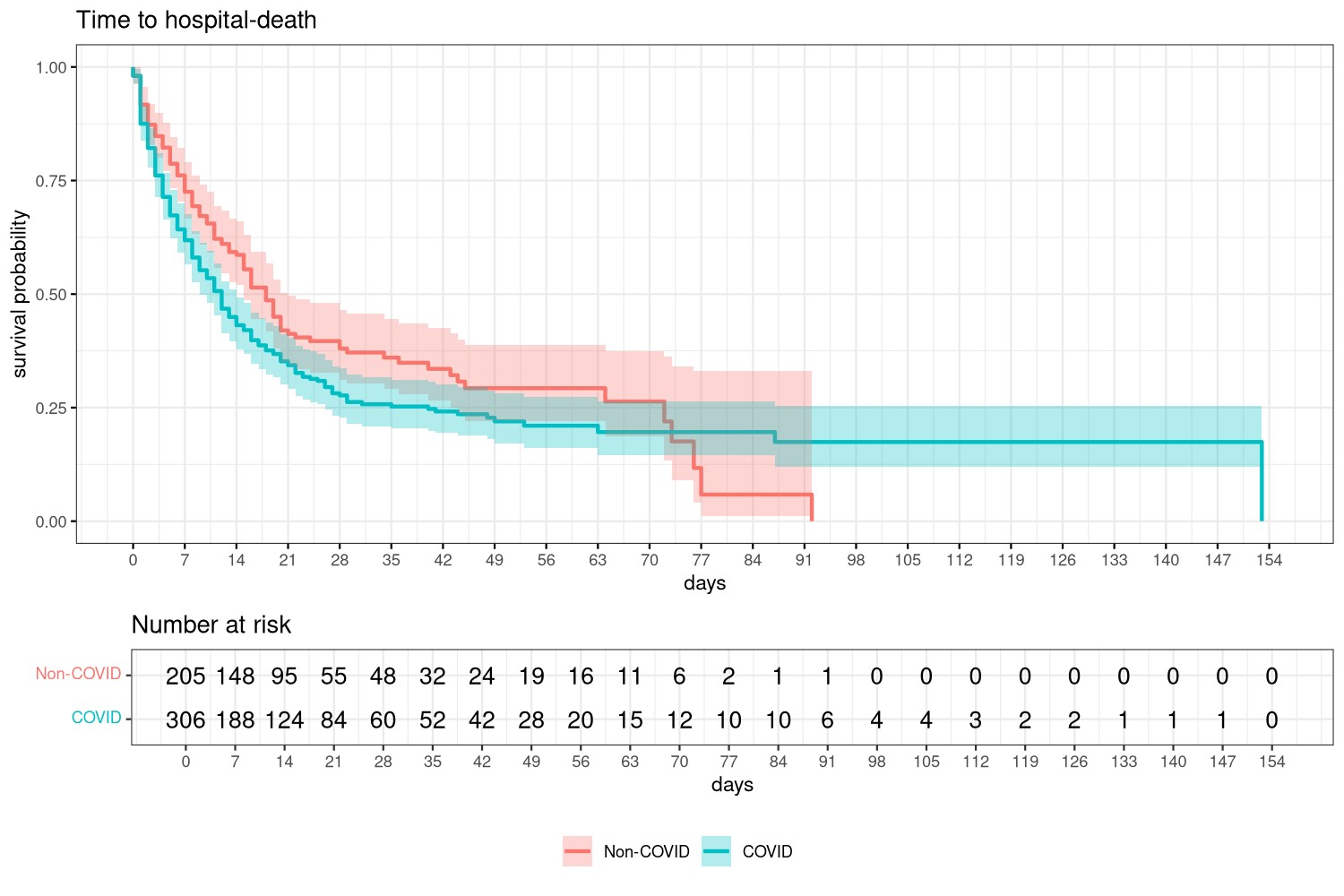

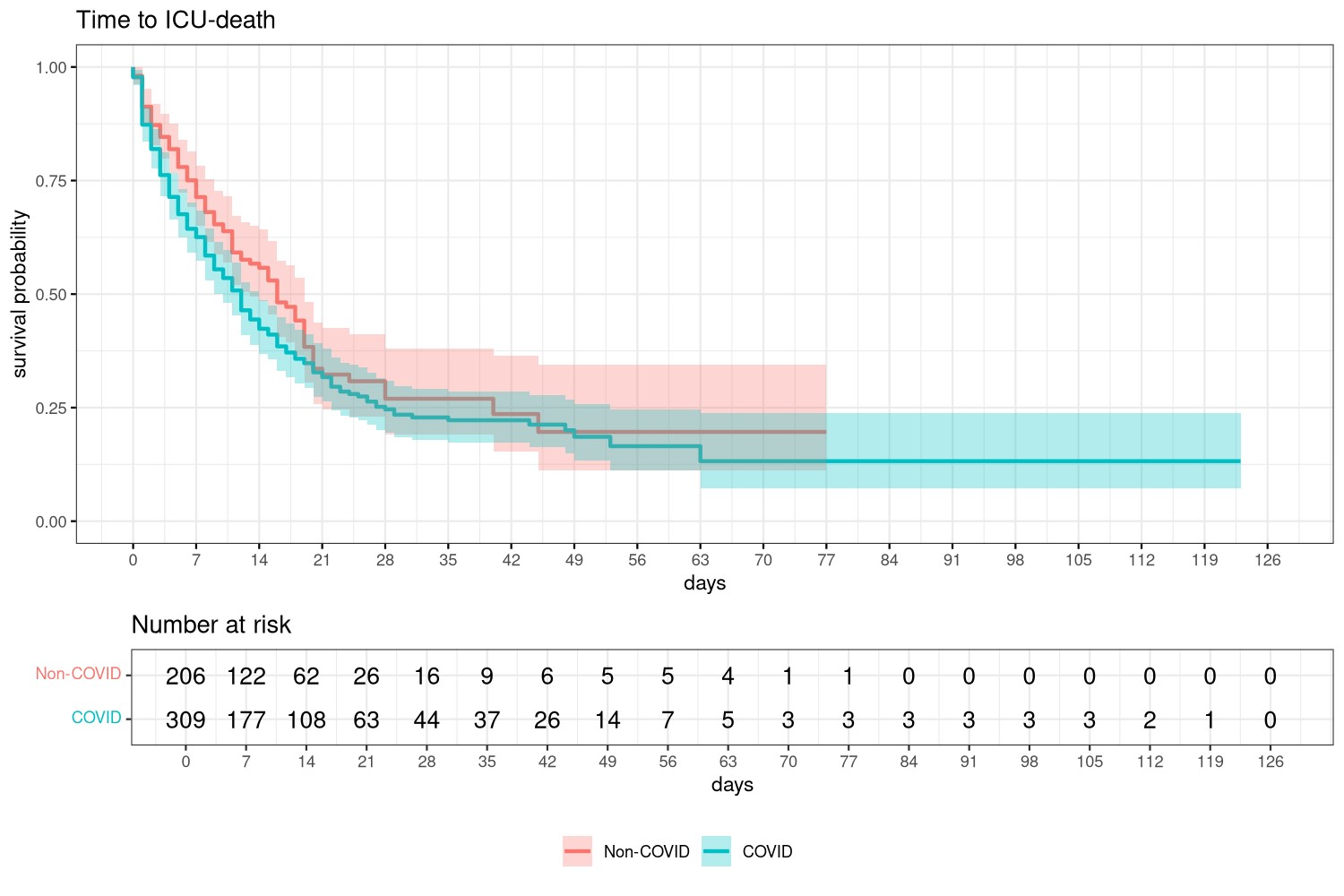

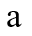

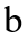


Legend: ICU – intensive care unit.
